# Water Adsorption at Pairs of Proximate Brønsted Acid Sites in Zeolites

**DOI:** 10.1021/acs.jpclett.5c03794

**Published:** 2026-01-14

**Authors:** Henning Windeck, Daniel Willimetz, Andreas Erlebach, Christopher J. Heard, Lukáš Grajciar, Fabian Berger, Joachim Sauer

**Affiliations:** † Department of Chemistry, 9373 Humboldt-University Berlin, 10099 Berlin, Germany; ‡ Department of Physical and Macromolecular Chemistry, 37740 Charles University, 12843 Prague, Czech Republic

## Abstract

We model water adsorption at pairs of proximate Brønsted acid sites (BASs) in zeolites H-MFI, H-FAU, and H-CHA. We use machine-learning potentials to explore the potential energy surface, combined with quantum mechanical methods for chemically accurate energies of selected structures. We identify BAS pairs that adsorb water cooperatively, forming an H-bonded chain that connects the two BASs and provides additional stabilization. The formation of such a water bridge requires at least two molecules, making the adsorption of the second water molecule stronger than the first, e.g., by 20 and 44 kJ mol^–1^ for an Al9–Al10 and an Al4–Al6 pair, respectively, in H-MFI, and by 11 kJ mol^–1^ for H-FAU. The magnitude of this extra-stabilization depends on the relative alignment of the BASs. Both Al pairs separated by just one SiO_4_ tetrahedron (next-nearest neighbor sites) and pairs across a 10-membered ring are included. The increase of the heat of adsorption with the water loading per BAS contrasts with the decrease obtained for isolated BASs and aligns with observations in some experiments.

## Graphical abstract

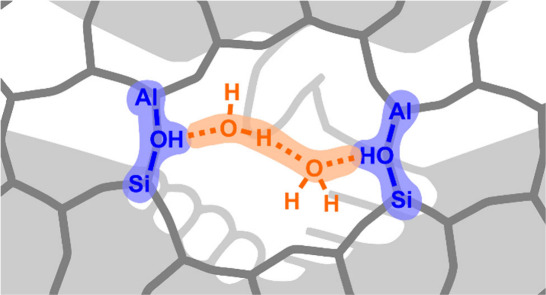

Acidic zeolites are porous crystalline aluminosilicates characterized by O_3_Si–O­(H)–AlO_3_ bridges, with OH groups which function as Brønsted acid sites (BASs).^1^ They have numerous applications, for example as catalysts for alkane conversion.^2^ Whereas traditional zeolite modeling typically considers idealized structures with isolated BASs,^3^ the effect of BAS pairs gets increasing attention^4–12^ as they can behave in strikingly different ways^7^ and modulate the reactivity and selectivity of catalytic reactions.

The amount and type of BAS pairs is given by the Al distribution in the framework which depends on the synthesis conditions^13^ rather than on the thermodynamic equilibrium between different possible distributions. Variation of the Al distribution can cause significant changes in acidity and catalytic activity.^7^ Assuming a random distribution, BAS pairs would be more abundant in zeolites with low rather than high Si/Al ratios, i.e., in zeolites with high rather than low Al concentrations. However, the abundance of BAS pairs can drastically differ between two zeolite samples with similar Si/Al ratios,^10^ questioning whether the Al distribution is truly random. Further, the Al atoms can be heterogeneously distributed in zeolite samples, forming Al-rich regions and parts essentially devoid of Al. This potentially allows for a local abundance of BAS pairs in zeolite samples with nominally high Si/Al ratios.^14^ Recently, a resonant soft X-ray scattering study suggested the location of a BAS pair in zeolite H-MFI and also showed that a BAS pair can cooperatively adsorb NH_3_ molecules.^15^

Water interacts strongly with BASs, thereby influencing the stability and reactivity of zeolite catalysts.^16–22^ Water is typically present when using feedstocks derived from biomass^23^ and important reactions produce water as a side product, e.g. alcohol dehydration. Previous studies of isolated BASs have shown that their adsorption strength for water strongly depends on their position in the framework.^24,25^ In this work, we study the effect of BAS pairs on the adsorption of water in Zeolite Socony Mobil-5 (H-MFI, H-ZSM-5), including also faujasite (H-FAU) and chabazite (H-CHA) as case studies. We aim to understand how different Al distributions influence water adsorption.

We combine two different computational modeling techniques: While machine learning interatomic potentials (MLIPs) enable long time-scale molecular dynamics (MD) simulations with nearly the accuracy of the method used for parametrization,^26–28^ quantum mechanical (QM) calculations can provide accurate adsorption energies for water^25,29,30^ and other adsorbates with strong H-bonds.^31,32^ Specifically, for the exploration of the potential energy surface (PES) via MD, we employ neural network potentials^33^ parametrized on dispersion-augmented density functional theory (DFT-D). The MLIP was trained on a comprehensive zeolite database, verified in previous works,^33,34^ and further validated using the uncertainty estimation approach developed by Willimetz et al.,^35^ see Section S2.1 in the Supporting Information for details. For calculating chemically accurate CCSD­(T)-level energies (CCSD­(T) - coupled cluster theory with singles, doubles, and perturbative triples substitutions)^36,37^ of selected structures, we employ a hybrid QM:QM method^29,38^ as applied before to water adsorption.^25^ It limits the high-level QM calculations to cluster models and adds long-range corrections calculated at the low-level with DFT-D, see Section S3.1 in the Supporting Information for details.

The orthorhombic MFI framework contains 12 crystallographically unique TO_4_ sites (T-sites, T = Si,Al), leading to many possible BAS configurations and pairs, which makes modeling of all of them computationally unfeasible. Our MLIP-based MD simulations show that in water-loaded systems the acidic proton is highly mobile, rapidly relocating between the O atoms of the AlO_4_ tetrahedron. This is consistent with previous findings^39,40^ that water clusters can deprotonate the BAS. Consequently, different BAS isomers are energetically nearly equivalent and accessible under dynamical conditions. Instead of considering every BAS isomer individually, the PES is globally sampled with the MLIP. Additional validation results are presented in Section S2 of the Supporting Information.

For the exploration of water adsorption on BAS pairs in H-MFI, we construct 16 models with Al atoms positioned at different framework sites, covering Al–Al distances ranging from 484 to 1024 pm. Section S1 of the Supporting Information presents all models, including an experimentally observed pair.^15^ For adsorption of one water molecule per BAS pair, MD simulations show that the water molecule remains localized at one of the BASs for all BAS pairs considered, i.e. the individual BASs within the BAS pair behave as isolated BASs. For adsorption of two water molecules per pair, for five of the BAS pairs, we find that each BAS binds one water molecule and the two water molecules are linked by an H-bond. We call them bridged BAS pairs (B) and all others non-bridged BAS pairs (NB), see Figure 1.

**Figure 1:**
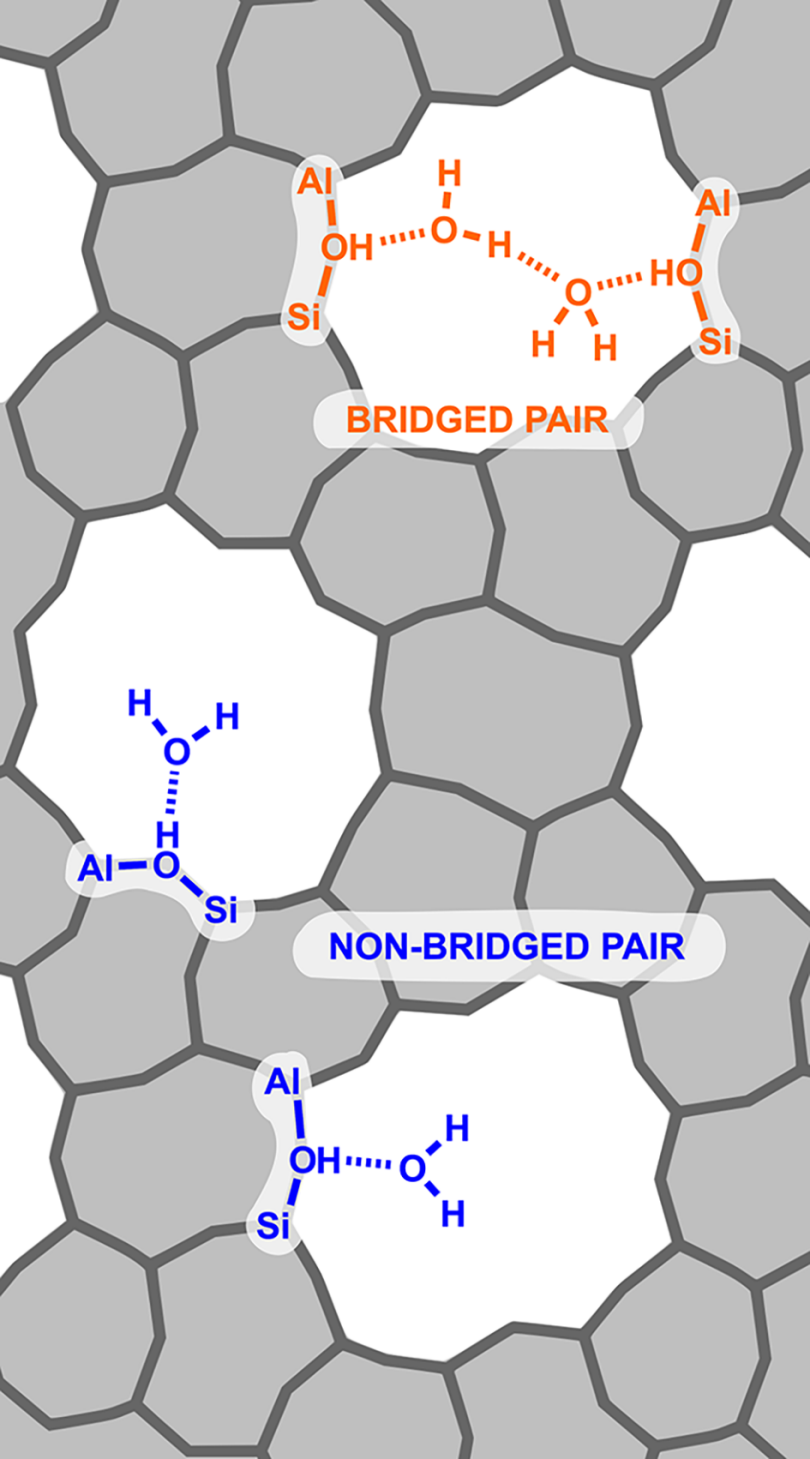
Scheme showing the difference between bridged (orange) and non-bridged (blue) BAS pairs.

We define the extra-stabilization gained when two water molecules adsorb at a BAS pair, Δ*E*_extra_, as the difference between the adsorption energy of both water molecules adsorbed at the BAS pair, *E*_ads_(2 H_2_O), and the sum of the adsorption energies of one water molecule at each site separately, *E*_ads_(1 H_2_O-A) and *E*_ads_(1 H_2_O-B):

$$\Delta E_{\mathrm{extra}}=E_{\mathrm{ads}}\left(2H_{2}O\right)-E_{\mathrm{ads}}\left(1H_{2}O\mathrm{‐A}\right)-E_{\mathrm{ads}}\left(1H_{2}O\mathrm{‐B}\right)$$ 1

Negative values indicate extra-stabilization resulting from cooperative water adsorption at a BAS pair. Figure 2 shows MP2 optimized adsorption structures of bridged BAS pairs. The B2 structure with Al at T10 and T3 shows protonation of the water dimer. As reported before,^25^ for the isolated BAS at T3, the protonated water dimer is more stable than the neutral one.

**Figure 2:**
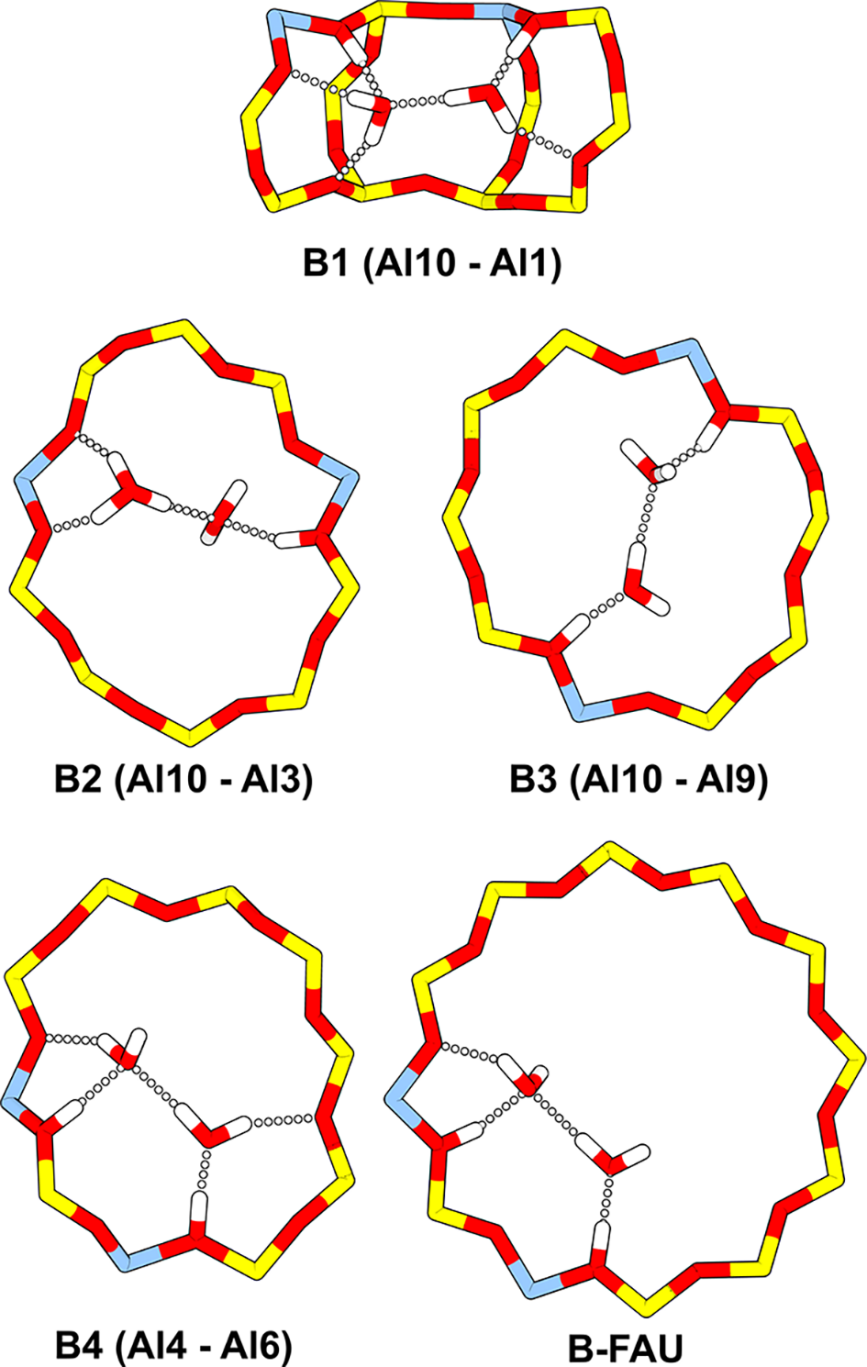
Optimized structures at MP2-level: Two water molecules bridging the B1, B2, B3, B4, and B-FAU pairs.

To analyze the origin of the extra-stabilization effect, we calculate the interaction energy (*E*_int_) and distortion energy (*E*_dis_) contributions to the adsorption energies as well as to the extra-stabilization energies (see also ref. 41). For adsorption of one water molecule, we define *E*_int_ as

$$E_{\mathrm{int}}=E_{Z-W}-\left(E_{W//Z-W}+E_{Z//Z-W}\right)$$ 2

where *E*_Z‑W_ is the energy of the zeolite-water complex. *E*_W//Z‑W_ and *E*_Z//Z‑W_ are the energies of the water molecule and the zeolite in the structure of the zeolite-water complex, respectively. For adsorption of two water molecules (W1 and W2), each water molecule enters with its own term:

$$E_{\mathrm{int}}=E_{Z-W1W2}-\left(E_{W1//Z-W1W2}+E_{W2//Z-W1W2}+E_{Z//Z-W1W2}\right)$$ 3

Finally, we calculate *E*_dis_ as the difference between *E*_ads_ and *E*_int_, based on the relation:

$$E_{\mathrm{ads}}=E_{\mathrm{int}}+E_{\mathrm{dis}}$$ 4

We screen all BAS pair models for extra-stabilization using average MD energies provided by the MLIP, see . Whereas some non-bridged BAS pairs exhibit minor extra-stabilization, a significant trend toward extra-stabilization is visible only for bridged BAS pairs, see . We select four bridged BAS pairs with significant extra-stabilization energy and calculate CCSD­(T)-quality adsorption energies. In addition, we consider two non-bridged BAS pairs to test whether they exhibit extra-stabilization at the CCSD­(T) level.

For the six selected BAS pairs, we extract the lowest-energy configurations from the MD trajectories and, as for water adsorption on isolated BASs,^25,42^ optimize these structures at the MP2-level (MP2 - second order Mo̷ller-Plesset perturbation theory).^43^ We employ the hybrid MP2:DFT-D approach^25,29,38,42^ with the Perdew Burke Ernzerhof (PBE)^44^ functional and the D2 dispersion term^45,46^ as the low-level method. As the final step, we calculate the CCSD­(T) – MP2 energy difference, ΔCC, for cluster models at the MP2 structure and approximate the CCSD­(T)-level adsorption energy as hybrid MP2:(PBE+D2)+ΔCC energy, hereafter called MP2+ΔCC. Table 1 presents the results for the selected BAS pairs.

**Table 1:** Al positions in Bridged (B) and Non-bridged (NB) BAS Pairs, Al–Al and H–H Distances (*R*, in pm) of the Bare Zeolite Structures Optimized at the MP2-Level^a^

		*R*		*E*_ads_ (*E*_int_/*E*_dis_)				
Pair	Position	Al–Al	H–H	1 H_2_O-A	1 H_2_O-B	2 H_2_O	2 H_2_O^b^	Δ*E*_extra_ (*E*_int_/*E*_dis_)
NB1	Al1–Al6	974	703	–77 (−105/28)	–78 (−111/33)	–152 (−209/57)	–76	3 (7/–4)
NB2	Al10–Al11	913	1249	–76 (−107/32)	–70 (−102/32)	–147 (−210/63)	–73	–1 (0/–1)
B1	Al10–Al1	615	475	–97 (−104/8)	–73 (−108/35)	–160 (−229/69)	–80	9 (−17/26)
B2	Al10–Al3	842	547	–78 (−93/15)	–77 (−114/37)	–160 (−/−)^c^	–80	–4 (−/−)^c^
B3	Al10–Al9	952	559	–80 (−112/32)	–76 (−107/30)	–176 (−218/41)	–88	–20 (1/–21)
B4	Al4–Al6	504	502	–54 (−104/49)	–57 (−102/45)	–156 (−199/43)	–78	–44 (7/–51)
								
B-FAU	Al1–Al1	603	417	–71 (−81/11)	–74 (−85/11)	–156 (−177/22)	–78	–11 (−11/0)

a MP2+ΔCC adsorption energies (*E*_ads_) and extra-stabilization energies (Δ*E*_extra_, see eq 1) in kJ mol^–1^. Contributions from interaction (*E*_int_) and distortion (*E*_dis_) energies as described in eqs 2–4.

b Per water molecule: *E*_ads_(2 H_2_O)/2.

c Deprotonation of BAS.

The MP2+ΔCC energies show that the formation of a water bridge between two BASs can indeed lead to significant extra-stabilization of −20 and −44 kJ mol^–1^ for the B3 and B4 bridged pairs, respectively. Decomposition of the extra-stabilization reveals that this effect originates from framework distortion, whereas the extra-interaction energies are as small as 1 and 7 kJ mol^–1^, respectively. Adsorption of the first water molecule induces significant framework distortion. The associated energy penalty can be significantly smaller for adsorption of a second water molecule. Thus, the first water molecule effectively prepares the framework, allowing the second to adsorb more strongly without a comparable penalty from framework distortion. For the non-bridged pairs NB1 and NB2, extra-stabilization values close to zero indicate that a water bridge is required for significant extra stabilization. The distortion energies for adsorption of one water molecule per site are similar as in the bridged pairs, but there is no cooperativity when the second water molecule is added. Whereas NB1 and NB2 feature Al–Al distances like B3, the BASs point into different pores. This indicates that the reduced distortion penalty for the second water molecule is a local effect. Despite formation of a water bridge, the extra-stabilization energy for the B1 pair is positive. This is due to an extra-distortion energy of 26 kJ mol^–1^ needed for adjusting the BAS structures at the Al10 and Al1 positions, which exceeds the extra-interaction energy of −17 kJ mol^–1^. The extra-stabilization is not a function of the Al–Al distance, and only BAS pairs with Al in specific positions support the formation of water bridges. Even BAS pairs with next-nearest neighbor Al atoms can behave like isolated BASs if their spatial orientation prevents favorable bridge formation. Notably, water adsorption strengths at each BAS within a pair cannot be predicted from those of the corresponding isolated BASs, see .

Further evidence for the role of water bridges as origin of extra-stabilization comes from MD simulations. For the six selected BAS pairs in H-MFI, Figure 3 shows the average number of H-bonds throughout the MD simulations performed at 350 K. For numerical results and distance criteria see . For all water adsorption structures, there are permanent H-bonds between the BAS and a water molecule, while weaker secondary H-bonds between the H atoms of the water molecule and different O_3_T-O-SiO_3_ (T = Si, Al) acceptor sites in the framework may form and break. For adsorption of 1 H_2_O/pair, all pairs form approximately two H-bonds, the first between the water molecule and one of the BASs and the second between the water molecule and varying O_3_T-O-SiO_3_ H-bond acceptors in the framework. For adsorption of 2 H_2_O/pair, however, a clear distinction emerges. Pairs that show negative Δ*E*_extra_ values feature on average around one H-bond more than others, 4.5–4.9 H-bonds compared to 3.7–3.9 for pairs without extra-stabilization. Furthermore, the bottom part of Figure 3 shows that the fraction of MD time a water bridge is present correlates with the extra-stabilization energy.

**Figure 3:**
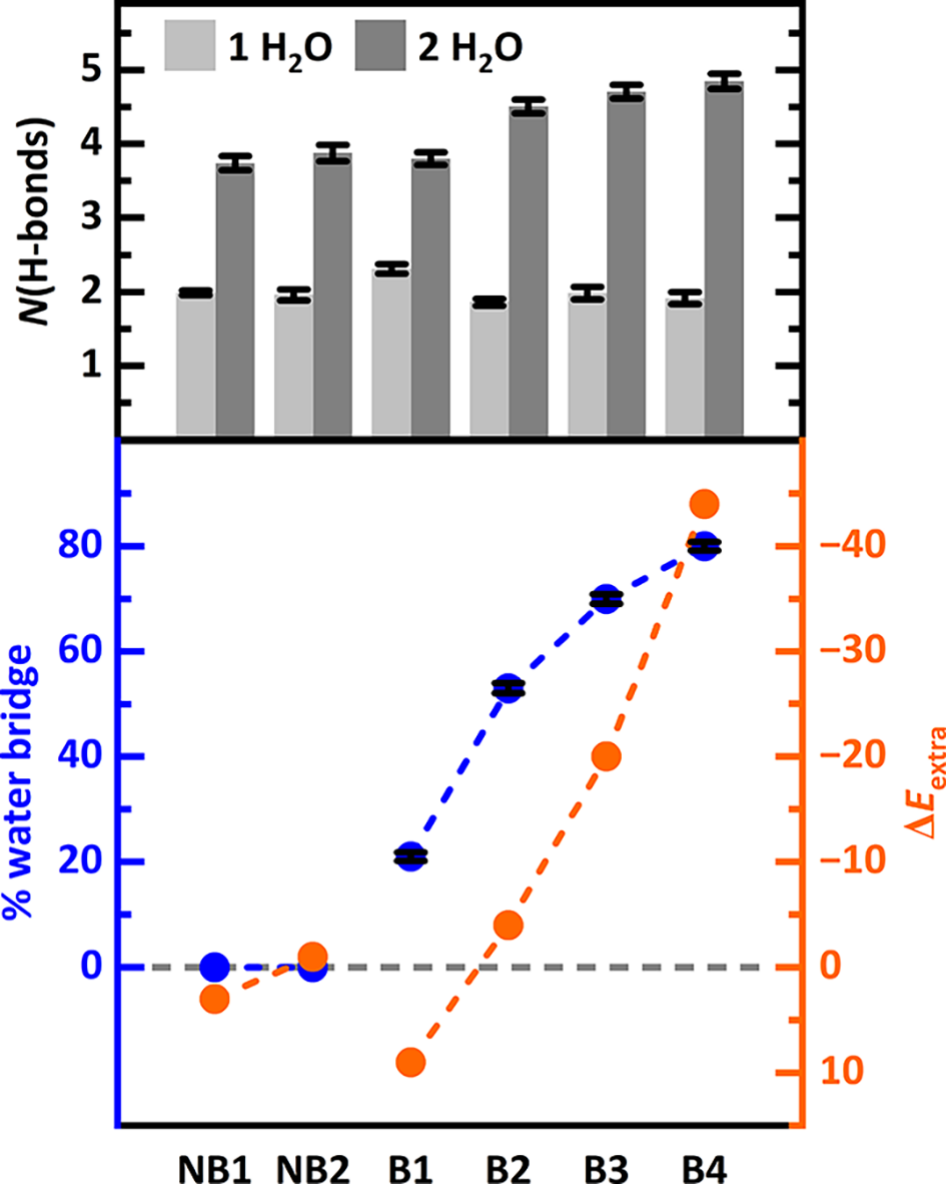
Average number of H-bonds throughout 1 ns MD simulations at 350 K for adsorption of one and two water molecules per BAS pair. Fraction of structures with a water bridge between the BASs in % (blue). MP2+ΔCC extra-stabilization energy (Δ*E*_extra_) in kJ mol^–1^ (orange). documents the procedure for calculation of error bars.

In addition to H-MFI, we investigate the widely used H-FAU and H-CHA zeolites with the same methodology, see . Their frameworks feature only one crystallographically unique Al site compared to 12 in orthorhombic MFI, which significantly reduces the modeling complexity. In zeolite H-FAU, we identify one BAS pair (B-FAU) with a water bridge and a negative Δ*E*_extra_ value of −11 kJ mol^–1^, see Table 1. In contrast to H-MFI, the extra-stabilization comes entirely from the interaction energy. The B-FAU adsorption structure is similar to the B4 structure, (see Figure 2). Due to the large pores of H-FAU, the adsorbed water molecules stay close to the BASs and do not interact with other parts of the framework. Thus, stable water bridges can only form for BAS pairs in immediate spatial proximity, i.e., those with one Si atom separating the Al atoms. The difference between the B-FAU and B4 structures is the ring-size, 12- compared to 10-membered rings of TO_4_ tetrahedra. While water bridging across a channel is not possible in H-FAU, it is possible across the smaller rings in H-MFI, as the B2 and B3 structures show. In contrast to H-FAU and H-MFI, for H-CHA, with its relatively small channels and large cages, we do not find any BAS pairs with extra-stabilization, i.e. favorable configurations of water bridges. This shows that the role of BAS pairs in water adsorption is highly sensitive to the zeolite topology.

Lercher and co-workers^47^ measured the heat of water adsorption as a function of water molecules per BAS for zeolite H-MFI. Surprisingly, they observed a significant increase in the adsorption heat with increasing loading between 0.5 and 1.5 H_2_O/BAS, see Figure 4. Previous work^25^ as well as our results presented in indicate that this behavior cannot be explained with uniform water adsorption at isolated, ideal BASs. The experiments yield average values for an (unknown) Al distribution and a nonuniform distribution of water molecules over the different types and locations of sites. The results of this work in Figure 4 show that cooperative water adsorption by BAS pairs does lead to an increase in the heat of adsorption from loading 1 H_2_O/pair (0.5 H_2_O/BAS) to loading 2 H_2_O/pair (1 H_2_O/BAS). Such pairs could contribute to the experimentally observed increase in adsorption heat.

**Figure 4:**
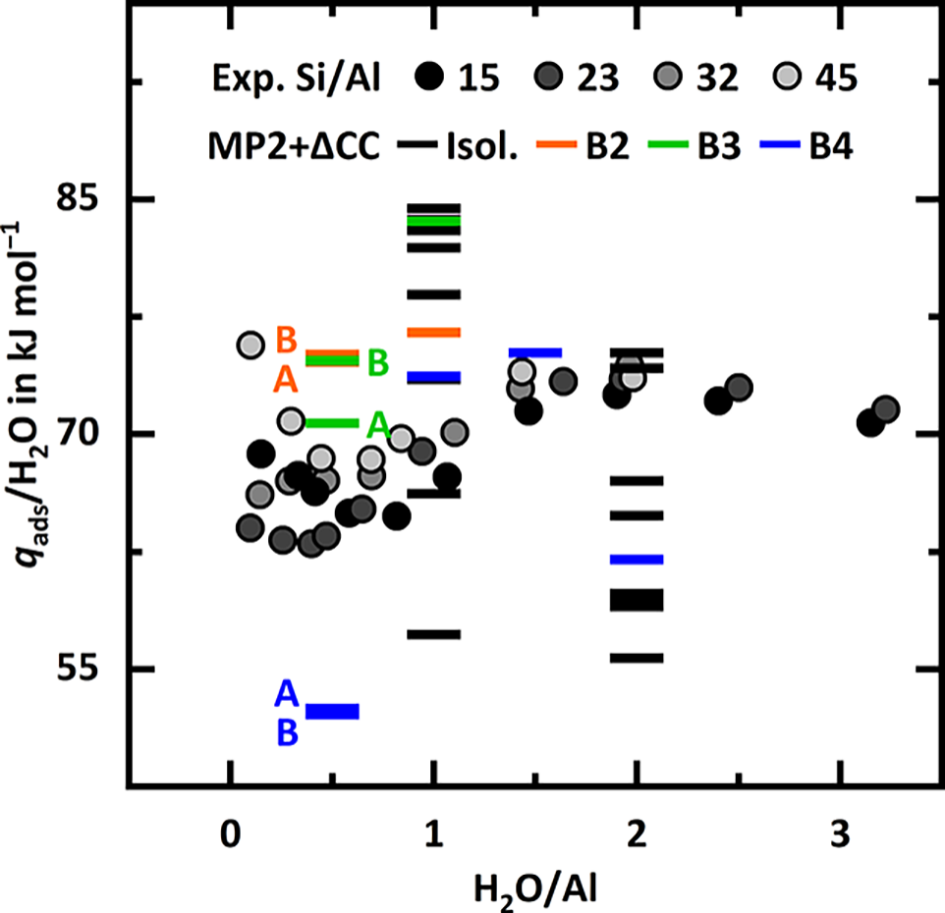
Experimental adsorption heats per H_2_O (*q*_ads_) in kJ mol^–1^ for samples with Si/Al ratios of 15, 23, 32, and 45.^47^ MP2+ΔCC adsorption enthalpies (absolute values) at 298 K for isolated BASs (black) as well as B2 (orange), B3 (green), and B4 (blue) pairs, see . For loading of 0.5 H_2_O/Al (1 H_2_O/pair), A and B refer to adsorption of one molecule at either of the two Al sites, 1 H_2_O-A and 1 H_2_O-B as introduced in Table 1.

Direct experimental evidence was recently reported for a BAS pair within the MFI framework between the T4 and T6 sites,^15^ following the IZA nomenclature.^48^ This specific pair (B4) exhibits a significant extra-stabilization of −44 kJ mol^–1^. For loading 0.5 H_2_O/BAS (1 H_2_O/pair), however, it exhibits a much weaker adsorption than most isolated BASs, see Figure 4. Thus, this pair is not expected to contribute significantly to the experimentally measured adsorption heats in the very low loading regime. Since the B4 pair shows relatively low adsorption heats for the first water molecule, we also investigate higher water loadings. We predict another increase in adsorption heat from 2 to 3 H_2_O/pair (1 to 1.5 H_2_O/BAS) followed by a decrease from 3 to 4 H_2_O/pair (1.5 to 2 H_2_O/BAS) which is compatible with the loading-dependent pattern of the experimental heats of adsorption.

The B3 pair (green symbols) exhibits both a significant extra-stabilization of −20 kJ mol^–1^ and relatively strong adsorption of the first water molecule compared to isolated BASs. The calculated data suggests that cooperative water adsorption at BAS pairs may contribute to the experimentally observed increase in adsorption heat.^47^ The adsorption heats calculated for specific sites and pairs can be much higher or lower than the experimental ones, which are an average observed for an ensemble of different acidic sites. Similar experiments as the ones of Lercher and co-workers^47^ on different H-MFI samples^49^ did not yield the same increase in adsorption heat in the low-loading regime. We compare these experimental results to our calculations in . Both the variations between experiments and the fairly large range of calculated adsorption heats for a given loading suggest that water adsorption is highly dependent on the specific sample and Al distribution.

We conclude that water adsorption at BAS pairs can differ significantly from that at isolated BASs. Combining the strengths of machine learning interatomic potentials and CCSD­(T) quality calculations, we have identified specific BAS pairs in H-MFI and H-FAU that adsorb two water molecules cooperatively. The molecules at the two BASs are bridged by an additional H-bond. As the adsorption of the second water molecule can be more exothermic than that of the first, such sites could contribute to the so far unexplained increase of the adsorption heat with the loading between 0.5 and 1.5 H_2_O/BAS.^47^ The magnitude of this extra-stabilization depends strongly on the crystallographic position of the two BASs constituting the pair, which underlines that the adsorption behavior of acidic zeolites cannot be reliably predicted without *a priori* knowledge of the Si/Al ratio and the specific Al distribution.

## Supplementary Material





## Notes

Computational methodology in detail with a detailed account of all results for isolated and paired BAS. (PDF)
Optimized structures for all models and sample inputs for all types of calculations. (ZIP)

## Γ

D.W.: Fraunhofer SCAI, Virtual Materials Design, 53757 Sankt Augustin, Germany

## #

F.B.: University of Cambridge, Yusuf Hamied Department of Chemistry, Cambridge CB2 1EW, UK.
