# Supplementary material for: Water Adsorption at Pairs of Proximate Brønsted Acid Sites in Zeolites
Source: J Phys Chem Lett. 2026 Jan 14;17(4):1201–6. doi: 10.1021/acs.jpclett.5c03794 (PMC12862806; doi:10.1021/acs.jpclett.5c03794)
Supplement: Supplementary file 1 [file jz5c03794_si_001.pdf]

Supporting Information

Water Adsorption at Pairs of Proximate Brønsted Acid Sites in Zeolites

Henning Windeck[a§], Daniel Willimetz[b§Γ], Andreas Erlebach[b], Christopher J. Heard[b], Lukáš Grajciar[b], Fabian Berger[a†], and Joachim Sauer[a*]*

[a] *Department of Chemistry, Humboldt-University Berlin, 10099 Berlin, Germany.*

[b] *Department of Physical and Macromolecular Chemistry, Charles University, 12843 Prague, Czech Republic.*

* Email: lukas.grajciar@natur.cuni.cz (Lukáš Grajciar) and js@chemie.hu-berlin.de (Joachim Sauer)

[§] *H.W. and D.W. contributed equally to this work.*

S1 Models

Initial structures are based on the orthorhombic silica MFI framework obtained from the IZA database.[1] To introduce Brønsted acid sites (BAS), one or two silicon atoms are substituted by aluminum, resulting in isolated or paired Al sites, respectively. A charge-compensating proton is placed on a neighboring framework oxygen, oriented toward the pore channel. Water molecules are then added by placing the oxygen atom 1.5 Å from the acidic proton. Details of the Al pairs in MFI used in this study are provided in Table S1. The same approach is used when modelling Al pairs in FAU and CHA zeolites. More information on the categorization and molecular dynamics is provided in Section S2.

The Al atoms in MFI were positioned to cover a wide range of Al–Al distances, exploring different relative arrangements, including aluminum atoms within the same channels and in different channels, to fully investigate the effect of Al pairs on extra-stabilization. After identifying the trends in extra-stabilization within the MFI framework, we attempted to reproduce the favorable Al configurations in the CHA and FAU frameworks. However, the combination of large cages and narrow channels in the CHA framework did not allow the formation of similar configurations that were found to be stable in MFI.

Table S1: Overview of all Al pairs in MFI with extra stabilization energies ΔE_{extra} (Eq. 1) obtained from average MD energies (Section S2) in kJ mol^{-1} and water behavior at two water molecules per Al pair. The R(Al-Al) is obtained as an average Al-Al distance in pm from the MD simulation.

Pair ID	BAS	R(Al-Al)	Category	ΔE_{extra}
1	T1O1 (A)<br>T7O1 (B)	589	Bridged	–5.4
2	T1O1 (A)<br>T5O1 (B)	852	Non-bridged	–8.7
3 (B1)	T1O1 (A)<br>T10O23 (B)	583	Bridged	–4.2
4 (NB1)	T1O1 (A)<br>T6O13 (B)	931	Non-bridged	–0.5
5 (NB2)	T10O23 (A)<br>T11O16 (B)	922	Non-bridged	–3.3
6	T11O16 (A)<br>T11O16 (B)	484	Non-bridged	23.2
7	T11O16 (A)<br>T10O3 (B)	1024	Non-bridged	0.7
8	T2O2 (A)<br>T9O19 (B)	500	Non-bridged	1.4
9	T7O18 (A)<br>T9O19 (B)	642	Non-bridged	7.9
10	T7O16 (A)<br>T11O16 (B)	499	Non-bridged	9.2
11	T7O16 (A)<br>T5O1 (B)	773	Non-bridged	4.3
12	T5O1 (A)<br>T7O16 (B)	535	Non-bridged	3.7
13 (B3)	T9O15 (A)<br>T10O23 (B)	943	Bridged	–6.4
14	T10O23 (A)<br>T12O8 (B)	927	Non-bridged	0.6
15 (B2)	T3O8 (A)<br>T10O3 (B)	858	Bridged	–3.4
16 (B4)	T4O11 (A)<br>T6O13 (B)	860	Bridged	–17.0

S2 Neural Network Potentials

S2.1 Benchmarking

Machine-learning interatomic potentials (MLIPs), and specifically, the presently used neural network potentials (NNPs), can accurately reproduce reference DFT data, while enabling efficient sampling of large configuration spaces. In this work, we employ an MLIP trained on a comprehensive zeolite dataset[2] using the density functional theory (DFT) approximation SCAN+D3(BJ).[2-4] The zeolite database contains a diverse set of proton-exchanged aluminosilicate frameworks interacting with water, covering multiple topologies, Si/Al ratios, and water loadings. Configurations were sampled from *ab initio* molecular dynamics simulations and calculated at the SCAN + D3(BJ) level, ensuring coverage of both equilibrium and high-energy structures relevant for zeolite–water interactions. Further details on the database composition and construction are provided in the work of Erlebach et al.[2] This model was previously validated, where molecular dynamics simulations using the MLIP reproduced structural and energetic features of reference DFT simulations with the same exchange–correlation functional and dispersion correction.[2,5] The energies obtained from the MLIP were compared against MP2+ΔCCSD(T) reference values using a test set of MFI structures with isolated Al sites taken from earlier work by some of us.[6] All structures are locally optimized with the given method to ensure consistent adsorption energies.

Furthermore, three MD simulations, one for each zeolite at 1 H_2O per Al (MFI B1, FAU 1, CHA 1), were analyzed for uncertainty using the kernel density estimation (KDE) approach for machine learning model uncertainty in materials modeling, as introduced by Willimetz et al..[7] All three simulations yielded similarity scores higher than 0.99, confirming that the configurations considered in this study remain within the interpolation regime of the MLIP and do not involve extrapolation.

Figure S1 compares the correlation between adsorption energies obtained with MP2+ ΔCCSD(T) and those computed using (a) SCAN+D3(BJ) DFT and (b) the MLIP. Both methods yield results in good agreement with the high-level reference. Notably, both the DFT and MLIP systematically overestimate adsorption energies at higher water loadings, consistent with previous findings that meta-GGA level DFT tends to overbind in such cases.[6]

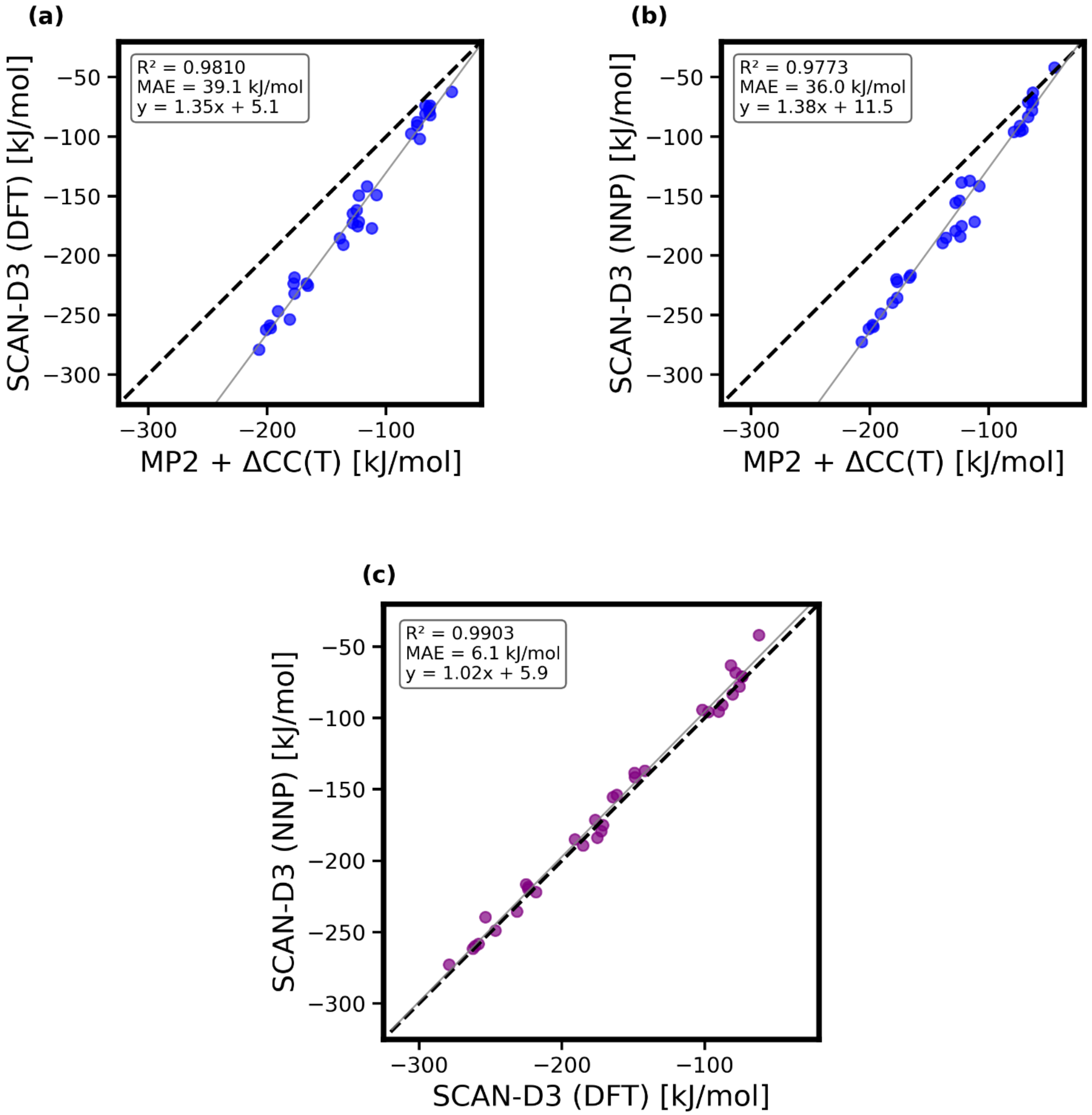

Figure S1: Correlation of adsorption energies in kJ mol^{-1} with MP2+ΔCC reference values for isolated Al sites in MFI. (a) Energies computed using SCAN+D3(BJ) DFT; (b) energies predicted using the neural network potential (MLIP); (c) correlation between SCAN+D3(BJ) and MLIP energies. The dashed line indicates perfect correlation, and gray line represents the linear fit. The mean absolute error (MAE) is the average absolute deviation between the MLIP-predicted energies and the reference energies.

Furthermore, MLIP can be further refined using the Δ-learning approach, in which a subset of the model parameters is frozen while the remaining parameters are optimized using a smaller dataset computed at a higher level of theory. We follow the same Δ-learning protocol as described by Erlebach et al.[2], from which the base MLIPs employed in this study are adopted. The identical strategy is applied to the MP2

reference data considered in this study. The Δ-learning approach cannot be applied to data obtained at the CC level of theory, as CC calculations do not provide forces by default. Moreover, the present dataset contains only 66 structures, which is well below the minimum recommended size of 150 structures reported by Erlebach et al. As a consequence, the resulting MLIPs exhibit test mean absolute errors exceeding 50 meV/atom for energies and 100 meV/Å for forces, rendering them unsuitable for further use in this work. These limitations could be potentially resolved by a more careful optimization of the training protocol and/or by increasing the size of the MP2 reference dataset, however, both approaches are beyond the scope of the present study.

S2.2 Molecular dynamics and structure selection

We use molecular dynamics (MD) simulations to explore the configurational space of considered zeolites (MFI, FAU, CHA). The simulations are driven by an MLIP developed by Erlebach et al.,[2] enabling efficient yet accurate sampling of thermally relevant structures.

Each system is simulated following a protocol consistent with previous work by some of us.[5] Simulations were carried out in the NVT ensemble for 1 ns using a time step of 0.5 fs. The temperature was maintained at 350 K using a Nosé–Hoover thermostat, and every 100th structure was saved for structural analysis. A radial cutoff of 6 Å was applied for interactions. MD simulations were performed using the Atomic Simulation Environment (ASE) version 3.22[8]. From the MD trajectories, the structure with the lowest predicted energy is identified and re-optimized using the same MLIP. This final structure serves as a starting point for the high-level MP2+ΔCC calculations. Since the autocorrelation time[9] is less than 1 ps for all MD simulations considered, the equilibration period was neglected, as the system reaches equilibrium almost immediately. Therefore, the average MD energy was computed over the entire simulation.

S2.3 Adsorption Energies

MD energies used to calculate adsorption energies are obtained as the average potential energy over 1 ns of MLIP-based MD simulation at 350 K. These values are reproducible and show minimal dependence on the initial configuration. In water-loaded zeolite frameworks, rapid proton hopping leads to energetic equilibration among different Brønsted acid site (BAS) isomers. As a result, it is not necessary to explicitly model all BAS configurations when evaluating adsorption at a specific T site with MD simulations.

S2.3.1 Isolated Sites at MFI

Total adsorption energies for MFI for models containing one aluminum atom per unit cell (Si/Al = 91) with one, two, or three water molecules per Brønsted acid site (BAS) are listed in Table S2. The variation in adsorption energies depending on the Al siting is consistent with our previous findings,[6] reflecting the structural complexity of the MFI framework.

Table S2: Total adsorption energies in kJ mol^{-1} for different water loadings, obtained from MLIP-MD simulations for MFI with Si/Al ratio of 91.

T site	1 H_2O	2 H_2O	3 H_2O
T1	−81	−165	−236
T2	−64	−144	−211
T3	−60	−145	−215
T4	−87	−163	−229
T5	−75	−168	−234
T6	−68	−149	−222
T7	−95	−167	−228
T8	−76	−153	−222
T9	−82	−166	−232
T10	−85	−160	−223
T11	−84	−162	−229
T12	−81	−169	−234

Monte Carlo simulations are performed to estimate the average adsorption energy as a function of water loading, treating the BAS in MFI as discrete lattice sites. For each water loading, an initial configuration is generated by randomly assigning water molecules to T-sites, with the constraint that no site hosts more than three molecules. Subsequent trial moves consist of randomly selecting a water molecule and attempting to transfer it to another site, with acceptance determined by a Boltzmann probability based on the difference in adsorption energies between the origin and destination sites (Metropolis criterion).[10] The simulations were implemented in a custom code written for this work and were performed in the canonical (NVT) ensemble at a temperature of 350 K. The simulation cell corresponds to the full MFI unit cell, including all T-sites, ensuring complete sampling of the framework. The Monte Carlo procedure was repeated until convergence of the average adsorption energy was reached, as determined using an autocorrelation analysis, with the standard error dropping well below 1 kJ mol^{-1}. In practice, this required approximately 1 million trial moves per water loading. The resulting ensemble-averaged energies and their standard deviations are plotted as a function of water molecules per BAS in Figure S2, providing a lattice Monte Carlo estimate of water adsorption behavior. This approach explicitly accounts for the variability in site energies across the different T-sites, while assuming a uniform Al distribution within the framework. The results show that the computed adsorption energies of isolated Al sites in MFI are not able to reproduce the experimental findings reported by Lercher et al., which is also supported by the conclusions of a previous study by some of us.[6]

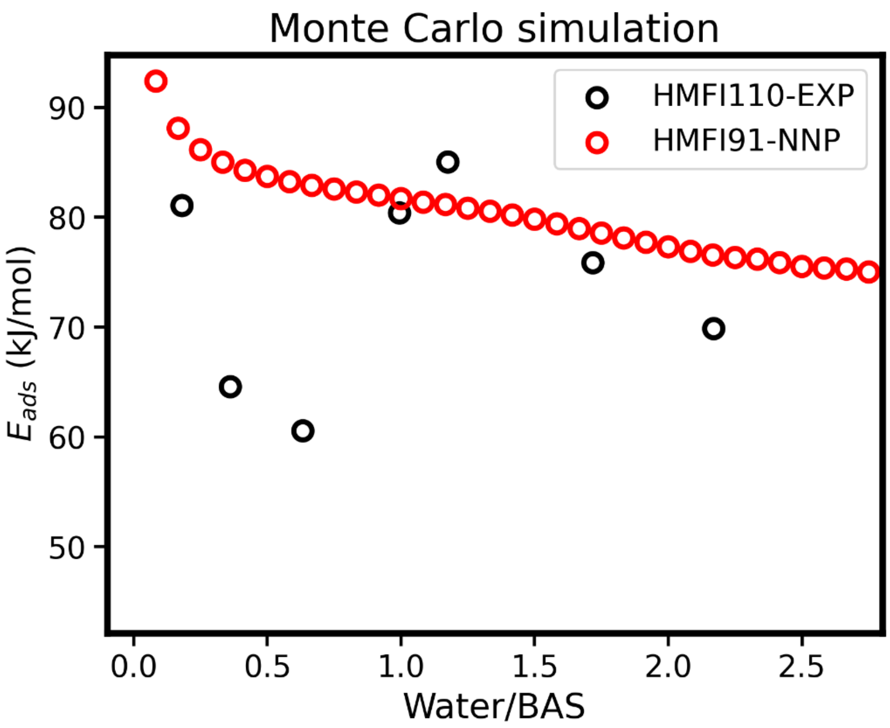

Figure S2: Monte Carlo simulation of water adsorption on isolated Al sites in MFI (Si/Al = 91). Each site is represented as a column of potential positions for up to three water molecules, and adsorption energies from Table S2 are used to model probabilistic site occupancy. Red circles indicate the simulated average adsorption energies per BAS as a function of water loading, while black circles show experimental values from Lercher and co-workers.[11]

Molecular dynamics simulations enable the investigation of dynamic properties such as proton solvation. A proton is considered solvated when the distance between a water molecule and the acidic proton is shorter than the distance between the proton and any framework oxygen atom. The solvation percentages for different water loadings are reported in Table S3.

Table S3: Proton solvation percentages at different water loadings and different T sites, as determined from molecular dynamics simulations.

T site	1 H_2O	2 H_2O	3 H_2O
T1	2%	88%	100%
T2	3%	85%	99%
T3	2%	85%	100%
T4	4%	79%	99%
T5	3%	95%	100%
T6	2%	82%	99%
T7	7%	90%	100%
T8	6%	83%	100%
T9	2%	88%	100%
T10	8%	84%	99%
T11	5%	82%	99%
T12	4%	86%	100%

As shown in Table S2, the solvation percentages for 1 and 2 water molecules per unit cell vary depending on the location of the Al atom. For systems with 3 water molecules, the proton is fully solvated. While the solvation percentage shows a weak correlation with the adsorption energies (Figure S3), this trend may be an artifact of the meta-GGA functional used to train the neural network potential.[6]

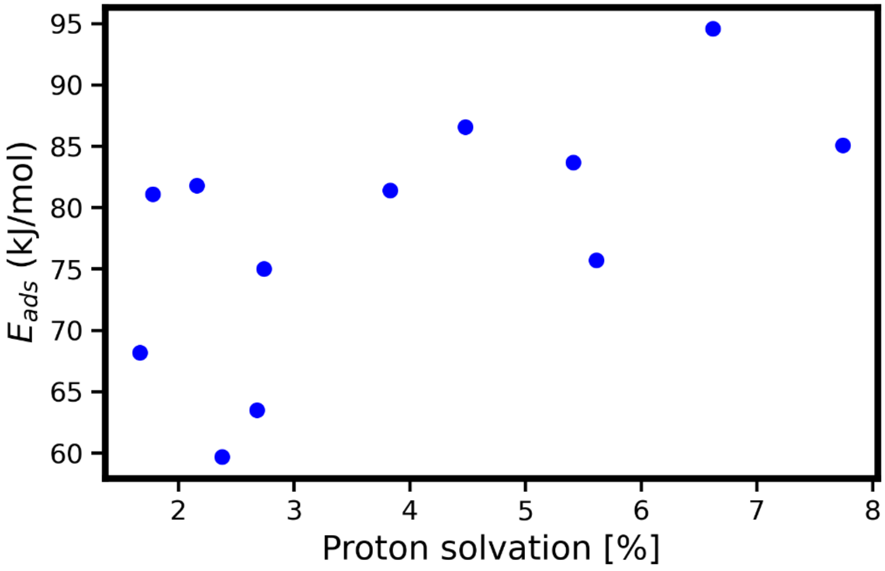

Figure S3: Correlation between proton solvation percentage and adsorption energy for systems with 1 water molecule per unit cell.

S2.3.2 Paired Sites at MFI

The individual adsorption energies of selected Al pairs are reported in Table S4. Due to the complexity of the MFI framework, Al pairs were initially generated at random and subsequently selected to form a representative set of four bridged and two non-bridged pairs, covering a wide range of Al–Al distances of up to ~10 Å. This small size of the set is necessary due to the computational demand of the MP2+ΔCC calculations.

Table S4: Average adsorption energies for representative Al pairs in MFI based on MLIP-MD simulations.

Pair ID	1 H_2O-A	1 H_2O-B	2 H_2O
B1	–83	–84	–172
NB1	–80	–77	–157
NB2	–85	–86	–174
B3	–92	–82	–180
B2	–89	–79	–171
B4	–87	–88	–192

Bridged Al pairs exhibit extra stabilization, caused by specific hydrogen-bonding motifs between water molecules and the zeolite framework. This effect can be quantitatively rationalized by counting hydrogen bonds, defined using a geometric description of an O–O distance below 2.4 Å[12] and an O–H···O angle greater than 110°.[13] A configuration is considered bridged when water molecules simultaneously form hydrogen bonds with each other and with framework oxygen atoms adjacent to both aluminum sites. The fractions of the molecular dynamics trajectories meeting these conditions are reported in Table S5.

To provide a graphical illustration of the molecular dynamics sampling, radial distribution functions (RDFs) were computed for two representative Al pairs in the MFI framework at a water loading of two water molecules: one bridged pair (MFI B2) and one non-bridged pair (MFI NB2). RDFs between Al atoms and oxygen atoms of the water molecules were calculated from the MLIP-MD trajectories and are shown in Figure S4. In the bridged case, pronounced peaks at short Al–O distances indicate that the water oxygen atoms are simultaneously close to their respective Al sites and to each other, consistent with the formation of a hydrogen-bonded water bridge. In contrast, such features are absent in the non-bridged configuration, confirming the absence of bridging interactions.

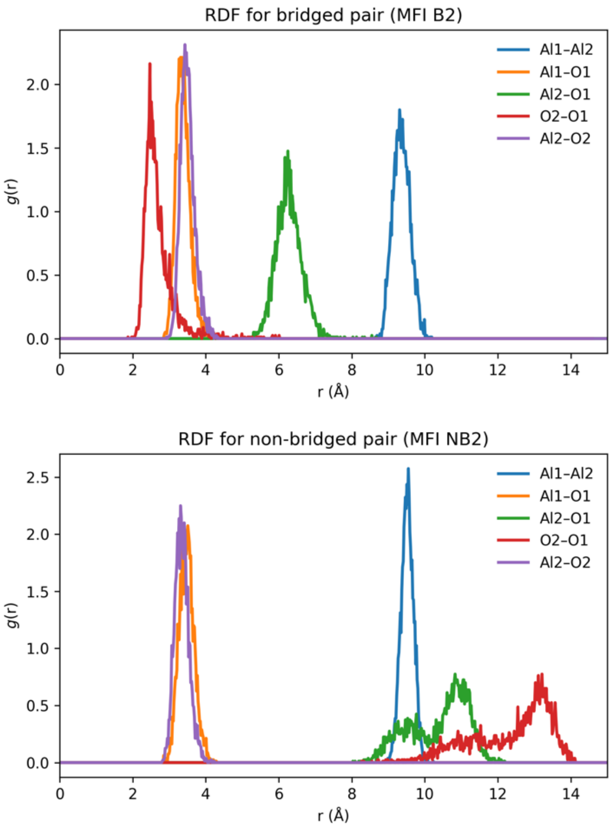

Figure S4: Radial distribution functions between Al atoms and oxygen atoms of water molecules for two representative Al pairs in the MFI framework at a water loading of two water molecules: a bridged pair (MFI B2) (top) and a non-bridged pair (MFI NB2) (bottom).

To evaluate the statistical significance of the MD results shown in Figure 3, we use the autocorrelation function of the hydrogen-bond time series to determine the autocorrelation time of the data. The total trajectory length was then divided by this correlation time to estimate the number of uncorrelated samples, which was used to determine the standard error of the mean.[14]

Table S5: The average number of hydrogen bonds in Al pairs evaluated for models containing one and two water molecules, along with the fraction of the simulation during which water molecules are bridging the BAS pair.

Pair ID	HB (1 H_2O)	HB (2 H_2O)	Bridged
B1	2.3	3.8	21%
NB1	2.0	3.7	0%
NB2	2.0	3.9	0%
B3	2.0	4.7	70%
B2	1.9	4.5	53%
B4	1.9	4.9	80%
FAU-1 (B-FAU)	1.4	4.0	95%
FAU-2	1.5	3.2	3%
CHA-1	1.9	3.8	0%
CHA-2	2.0	4.0	1%

The MFI framework features stabilizing Al pairs with favorable Al–Al distances and spatial arrangements that enable the formation of hydrogen-bonded water networks. The water adsorption configurations can be categorized as non-bridged (each BAS binds one water molecule or a single BAS binds two water molecules), or bridged (water molecules bridge two BAS sites). This classification is based on the solvation environment at each BAS, which differs between loadings of 1 and 2 water molecules, as reflected in the solvation percentages (Table S3) and the average number of hydrogen bonds (Table S5). This separation can be further validated by visually inspecting the MD trajectory. The categorization of all 16 MFI pairs is in Table S1. The hypothesis that bridged pairs exhibit additional stabilization is supported by a Fisher's exact test[15] ($p = 0.01$), confirming the statistical significance of the observation.

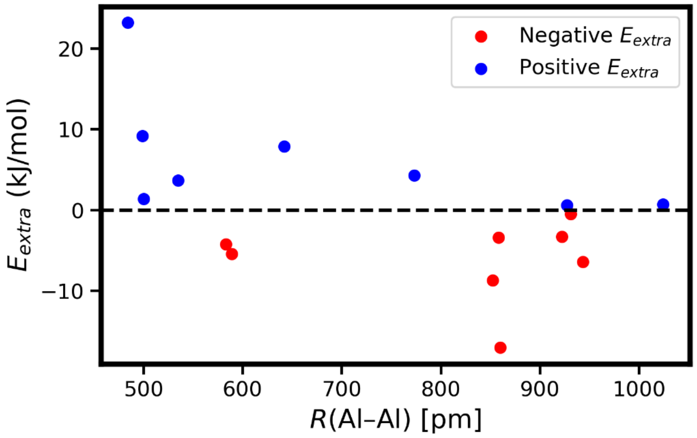

Figure S5: Correlation between Al-Al distance and the extra stabilization for 16 MFI pairs (Table S1).

To further illustrate the complexity of water-mediated stabilization, Figure S5 shows the correlation between the Al–Al distance in MFI pairs and the additional stabilization energy. While a moderate monotonic correlation is observed, stabilizing bridged pairs can occur even at short Al–Al distances (<600 pm), leading to substantial stabilization. However, most close Al pairs tested are not favorable for water bridging and instead result in slight destabilization.

S2.3.3 Paired sites at FAU and CHA

For the CHA and FAU zeolites, two distinct Al pairs were constructed within each framework, differing in either Al–Al distance or spatial arrangement. Due to the high symmetry and limited geometric flexibility of these frameworks, constructing additional Al pairs would either produce symmetrically equivalent environments or place the Al atoms too far apart to enable interaction via shared water molecules. The corresponding adsorption energies are summarized in Table S6. In the case of FAU, the framework geometry has been found to favor stabilizing water bridging. Notably, the FAU (1) pair remains bridged for most of the MD simulation (Table S5).

Table S6: Adsorption energies kJ mol^{-1} obtained from MLIP-MD average energies for selected aluminum pairs of FAU and CHA zeolites with Al-Al distance in pm.

Pair ID	R(Al-Al)	1 H_2O-A	1 H_2O-B	2 H_2O	Stabilization
FAU-1 (B-FAU)	601	−72	−73	−166	−21
FAU-2	803	−90	−87	−172	5
FAU-sodalite	810	−88	−85	−161	12
CHA-1	971	−97	−101	−190	8
CHA-2	930	−96	−92	−183	5

To further verify the sampling approach used in this study, we analyzed two structures at a water loading of two water molecules for pair 2 in H-CHA. One structure was obtained using the described protocol by selecting the lowest-energy configuration from the MD simulation, while the second corresponds to the lowest-energy structure with a bridge formation. The corresponding extra-stabilization energies computed at the MP2:(PBE+D2)+ΔCC level are 9 and 20 kJ mol^{-1}, respectively. This comparison demonstrates that the sampling approach reliably selects low-energy, representative structures. Furthermore, it confirms that if a bridged BAS pair provides extra-stabilization, such configurations would be identified by the sampling procedure.

S3 Quantum Chemical Results: Hybrid MP2:(PBE+D2)+ΔCC Approach

S3.1 Computational Details

For periodic DFT calculations, we use the PBE[16] exchange correlation functional augmented Grimme's D2 dispersion term[17] and Ewald summation[18] as implemented in VASP 5.4.1,[19-22] using plane wave basis sets for the valence electrons and the projector augmented wave (PAW) method for core electrons (standard potentials). We sample the Brillouin-zone at the Γ-point only. These settings proved suitable in studies on similar systems.[23-27] As the D2 and D3 dispersion corrections work equally well for the systems studied here, we remain with the former.[27] We use a kinetic energy cutoff of 400 eV. We use convergence thresholds of 10^{-7} eV for energy changes and 0.001 eV Å^{-1} for structure changes. We numerically calculate Hessian matrices using the central finite differences method with Cartesian displacements of 0.01 Å and a tighter convergence threshold of 10^{-8} eV for energy changes. Using the Hessians, we confirm stationary points by normal mode analysis and obtain zero-point vibrational energies as well as thermal enthalpy contributions.

We use a mechanical embedding scheme introduced and extensively tested before.[6,28-31] Using the MonaLisa code,[32] we couple MP2[33,34] and CCSD(T)[35,36] calculations on cluster models with PBE+D2 calculations on the periodic system. We perform cluster calculations with ORCA 4.2.1[37] and periodic calculations with VASP 5.4.1.[19-22] We saturate dangling bonds with H atoms with a fixed O-H distance of 0.953 Å.[29,30] We use the def2-TZVP basis set[38] for PBE+D2 low-level cluster calculations. In MP2:PBE+D2 structure optimizations, we use the def2-TZVPP basis set[38] for RI-MP2 high-level cluster calculations (RI=resolution of identity[39]). We provide cif-files with all employed cluster models as part of the Supporting Information.

We make sure that clusters are cut such that direct, strong H-bonding between the adsorbed water molecules and the OH groups at the periphery of the clusters is not possible. Weak interactions between water and the OH groups at the periphery of the clusters may occur. This is a consequence of an approach that uses cluster models. We do not use these clusters to calculate adsorption energies directly but rather to calculate a correction to the low-level method. The existence of such weak interactions would only take effect if the low-level and high-level methods describe them differently. We expect the corrections for weak, spurious interactions to be very small and thus to have only a very small effect on the final QM:QM adsorption energies. This is evidenced by previous QM:QM studies which reliably reached chemical accuracy using similarly sized (or smaller) cluster models.[28]

To obtain benchmark energies, we also add CCSD(T) corrections (ΔCC) to obtain single-point MP2:(PBE+D2)+ΔCC energies on our MP2:(PBE+D2) structures. For these single-point calculations, we use the domain-based local pair natural orbital (DLPNO) approximation[40,41] for MP2 and CCSD(T), employing the tightPNO settings. We use significantly larger cluster models in these single-point calculations than in the structure optimizations. Further, we extrapolate MP2 and CCSD(T) energies to the complete basis set limit with cc-pV*n*Z basis sets[42,43] in a two-point extrapolation scheme[44,45] and apply

the counterpoise correction to counteract the basis set superposition error.[46] To obtain adsorption enthalpies (H_{ads}), we add PBE+D2 level zero-point vibrational energies and thermal corrections, as given by

$$H_{ads} = E_{ads} + E_{ZPE} + H_{0K\rightarrow 298K} - RT \quad \text{(S1)}$$

where E_{ads} corresponds to MP2:(PBE+D2)+ΔCC electronic adsorption energies, E_{ZPE} refers to PBE+D2 zero-point vibrational energies, $H_{0K\rightarrow 298K}$ to PBE+D2 thermal corrections at 298 K, T to the temperature, and R to the universal gas constant.

S3.2 Water Adsorption in H-MFI

Table S7 shows the PBE+D2 results for the six selected BAS pairs. Table S8 shows the benchmark MP2:(PBE+D2)+ΔCC results for them, including also the MP2 and CCSD(T) corrections. Table S9 and S10 show the results for the isolated BAS that correspond to the BAS in the pairs as obtained with PBE+D2 and MP2:(PBE+D2)+ΔCC, respectively. Figure S6 compares extra stabilization energies from average and lowest MD energies as well as from MP2:(PBE+D2)+ΔCC. Figure S7 visually compares the adsorption energies for BAS in a pair and the corresponding isolated BAS, demonstrating that they are uncorrelated. Figure S8 compares our results to the measurements of Olson and co-workers.[47]

Table S7: For H-MFI, PBE+D2 adsorption energies (E_{ads}) and enthalpies at 298 K (H_{ads}) with zero-point vibrational energies (ΔZPE) and thermal contributions ($\Delta H_{0K\rightarrow298K}$) as well as extra-stabilization energies (ΔE_{extra}) and enthalpies (ΔH_{extra}) in kJ mol^{-1}, see Eq. 1. Structures optimized with PBE+D2.

Pair ID	Loading	ΔZPE	$\Delta H_{0K\rightarrow298K}$	E_{ads}	E_{ads}/H_2O	H_{ads}	H_{ads}/H_2O	ΔE_{extra}	ΔH_{extra}
NB1	1 H_2O-A	8.2	−6.0	−96.9	−96.9	−94.7	−94.7	–	–
	1 H_2O-B	8.6	−6.2	−98.2	−98.2	−95.8	−95.8	–	–
	2 H_2O	17.5	−10.5	−186.2	−93.1	−179.2	−89.6	9.0	11.3
NB2	1 H_2O-A	3.0	−1.8	−90.3	−90.3	−89.1	−89.1	–	–
	1 H_2O-B	4.9	−3.6	−89.7	−89.7	−88.4	−88.4	–	–
	2 H_2O	16.2	−10.1	−184.7	−92.3	−178.7	−89.3	−4.8	−1.1
B1	1 H_2O-A	7.5	−5.6	−112.7	−112.7	−110.8	−110.8	–	–
	1 H_2O-B	10.9	−5.7	−92.1	−92.1	−86.9	−86.9	–	–
	2 H_2O	21.1	−13.5	−201.6	−100.8	−194.0	−97.0	3.2	3.7
B2	1 H_2O-A	7.7	−4.4	−94.0	−94.0	−90.7	−90.7	–	–
	1 H_2O-B	9.9	−7.2	−99.2	−99.2	−96.6	−96.6	–	–
	2 H_2O	17.4	−10.8	−208.0	−104.0	−201.5	−100.7	−14.8	−14.1
B3	1 H_2O-A	12.0	−7.0	−99.2	−99.2	−94.2	−94.2	–	–
	1 H_2O-B	12.3	−6.8	−92.1	−92.1	−86.6	−86.6	–	–
	2 H_2O	19.6	−10.6	−210.7	−105.4	−201.7	−100.8	−19.4	−20.8
B4	1 H_2O-A	7.8	−5.5	−82.0	−82.0	−79.7	−79.7	–	–
	1 H_2O-B	10.4	−5.6	−79.4	−79.4	−74.6	−74.6	–	–
	2 H_2O	17.7	−9.2	−182.3	−91.1	−173.8	−86.9	−20.9	−19.5
	3 H_2O	31.1	−17.8	−301.5	−100.5	−288.2	−96.1	–	–
	4 H_2O	39.7	−21.4	−349.5	−87.4	−331.2	−82.8	–	–

Table S8: For H-MFI, MP2:(PBE+D2)+ΔCC adsorption energies (E_{ads}) and enthalpies at 298 K (H_{ads}) with corrections from MP2 (ΔMP2) and CCSD(T) (ΔCC) as well as extra-stabilization energies (ΔE_{extra}) and enthalpies (ΔH_{extra}) in kJ mol^{-1}, see Eq. 1. Structures optimized with MP2:PBE+D2. Enthalpies calculated with zero-point vibrational energies (ΔZPE) and thermal contributions ($\Delta H_{0K\rightarrow298K}$) from PBE+D2, see Table S7.

Pair ID	Loading	ΔMP2	ΔMP2/ H_2O	ΔCC	ΔCC/ H_2O	E_{ads}	E_{ads}/ H_2O	H_{ads}	H_{ads}/ H_2O	ΔE_{extra}	ΔH_{extra}
NB1	1 H_2O-A	16.9	16.9	1.0	1.0	−77.1	−77.1	−74.9	−74.9		
	1 H_2O-B	17.6	17.6	0.8	0.8	−77.7	−77.7	−75.3	−75.3		
	2 H_2O	28.6	14.3	2.6	1.3	−151.8	−75.9	−144.8	−72.4	3.1	5.4
NB2	1 H_2O-A	11.3	11.3	1.4	1.4	−75.8	−75.8	−74.6	−74.6		
	1 H_2O-B	16.4	16.4	1.5	1.5	−70.0	−70.0	−68.8	−68.8		
	2 H_2O	32.5	16.3	2.0	1.0	−146.7	−73.4	−140.7	−70.3	−0.9	2.7
B1	1 H_2O-A	13.5	13.5	0.5	0.5	−96.9	−96.9	−95.0	−95.0		
	1 H_2O-B	17.4	17.4	1.0	1.0	−72.7	−72.7	−67.5	−67.5		
	2 H_2O	34.8	17.4	3.5	1.7	−160.5	−80.2	−152.8	−76.4	9.1	9.7
B2	1 H_2O-A	12.5	12.5	1.1	1.1	−78.4	−78.4	−75.1	−75.1		
	1 H_2O-B	17.9	17.9	1.6	1.6	−77.2	−77.2	−74.6	−74.6		
	2 H_2O	40.2	20.1	5.8	2.9	−159.6	−79.8	−153.1	−76.5	−3.9	−3.3
B3	1 H_2O-A	16.6	16.6	0.7	0.7	−79.7	−79.7	−74.7	−74.7		
	1 H_2O-B	13.8	13.8	0.7	0.7	−76.1	−76.1	−70.7	−70.7		
	2 H_2O	29.4	14.7	1.6	0.8	−176.2	−88.1	−167.2	−83.6	−20.4	−21.8
B4	1 H_2O-A	23.9	23.9	1.7	1.7	−54.4	−54.4	−52.1	−52.1		
	1 H_2O-B	18.7	18.7	1.1	1.1	−57.3	−57.3	−52.5	−52.5		
	2 H_2O	21.0	10.5	1.9	0.9	−156.0	−78.0	−147.5	−73.7	−44.3	−42.9
	3 H_2O	52.0	17.3	6.3	2.1	−238.9	−79.6	−225.7	−75.2		
	4 H_2O	69.5	17.4	8.3	2.1	−266.4	−66.6	−248.1	−62.0		

Table S9: For isolated BAS in H-MFI corresponding to the BAS pairs in Table S7, PBE+D2 adsorption energies (E_{ads}) and enthalpies at 298 K (H_{ads}) with zero-point vibrational energies (ΔZPE) and thermal contributions ($\Delta H_{0K\rightarrow298K}$) as well as extra-stabilization energies (ΔE_{extra}) and enthalpies (ΔH_{extra}) in kJ mol^{-1}, see Eq. 1. Structures optimized with PBE+D2.

Position	Loading	ΔZPE	$\Delta H_{0K\rightarrow298K}$	E_{ads}	E_{ads}/H_2O	H_{ads}	H_{ads}/H_2O
Al10-O23(H)-Si10	1 H_2O	5.1	−3.6	−102.8	−102.8	−101.3	−101.3
	2 H_2O	20.4	−9.8	−183.6	−91.8	−173.1	−86.5
Al10-O3(H)-Si1	1 H_2O	0.0	−3.2	−107.6	−107.6	−110.8	−110.8
	2 H_2O	10.7	−5.2	−189.0	−94.5	−183.4	−91.7
Al11-O16(H)-Si7	1 H_2O	11.3	−5.6	−80.2	−80.2	−74.5	−74.5
	2 H_2O	20.9	−10.4	−173.9	−87.0	−163.4	−81.7
Al1-O1(H)-Si5	1 H_2O	8.4	−4.8	−93.3	−93.3	−89.6	−89.6
	2 H_2O	18.0	−11.0	−169.2	−84.6	−162.1	−81.1
Al3-O8(H)-Si12	1 H_2O	8.2	−4.6	−99.7	−99.7	−96.1	−96.1
	2 H_2O	20.9	−11.3	−171.8	−85.9	−162.2	−81.1
Al4-O11(H)-Si7	1 H_2O	7.5	−3.2	−96.0	−96.0	−91.8	−91.8
	2 H_2O	12.8	−8.7	−197.0	−98.5	−192.8	−96.4
Al6-O13(H)-Si5	1 H_2O	12.6	−5.9	−86.0	−86.0	−79.3	−79.3
	2 H_2O	20.6	−11.0	−164.7	−82.3	−155.0	−77.5
Al9-O15(H)-Si6	1 H_2O	7.8	−4.6	−97.6	−97.6	−94.4	−94.4
	2 H_2O	14.2	−8.2	−186.9	−93.4	−180.8	−90.4

Table S10: For isolated BAS in H-MFI corresponding to the BAS pairs in Table S8, MP2:(PBE+D2)+ΔCC adsorption energies (E_{ads}) and enthalpies at 298 K (H_{ads}) with corrections from MP2 (ΔMP2) and CCSD(T) (ΔCC) as well as extra-stabilization energies (ΔE_{extra}) and enthalpies (ΔH_{extra}) in kJ mol^{-1}, see Eq. 1. Structures optimized with MP2:PBE+D2. Enthalpies calculated with zero-point vibrational energies (ΔZPE) and thermal contributions ($\Delta H_{0K\rightarrow 298K}$) from PBE+D2, see Table S9.

	Loading	ΔMP2	ΔMP2/ H_2O	ΔCC	ΔCC/ H_2O	E_{ads}	E_{ads}/ H_2O	H_{ads}	H_{ads}/ H_2O
Al10-O23(H)-Si10	1 H_2O	14.3	14.3	0.6	0.6	−85.9	−85.9	−84.4	−84.4
	2 H_2O	37.2	18.6	4.5	2.3	−140.2	−70.1	−129.7	−64.8
Al10-O3(H)-Si1	1 H_2O	21.0	21.0	4.5	4.5	−79.8	−79.8	−83.0	−83.0
	2 H_2O	28.4	14.2	5.3	2.6	−153.9	−77.0	−148.4	−74.2
Al11-O16(H)-Si7	1 H_2O	14.6	14.6	0.7	0.7	−62.9	−62.9	−57.2	−57.2
	2 H_2O	38.4	19.2	3.6	1.8	−129.7	−64.9	−119.3	−59.6
Al1-O1(H)-Si5	1 H_2O	13.0	13.0	1.5	1.5	−77.1	−77.1	−73.5	−73.5
	2 H_2O	34.3	17.1	6.1	3.0	−126.6	−63.3	−119.5	−59.8
Al3-O8(H)-Si12	1 H_2O	13.7	13.7	2.0	2.0	−82.5	−82.5	−78.9	−78.9
	2 H_2O	35.4	17.7	6.1	3.0	−127.6	−63.8	−118.0	−59.0
Al4-O11(H)-Si7	1 H_2O	4.5	4.5	1.5	1.5	−87.9	−87.9	−83.7	−83.7
	2 H_2O	35.2	17.6	4.8	2.4	−154.5	−77.3	−150.4	−75.2
Al6-O13(H)-Si5	1 H_2O	14.4	14.4	0.8	0.8	−72.8	−72.8	−66.2	−66.2
	2 H_2O	35.9	17.9	5.8	2.9	−121.0	−60.5	−111.3	−55.7
Al9-O15(H)-Si6	1 H_2O	10.0	10.0	0.6	0.6	−85.1	−85.1	−81.9	−81.9
	2 H_2O	39.4	19.7	4.6	2.3	−140.1	−70.0	−134.0	−67.0

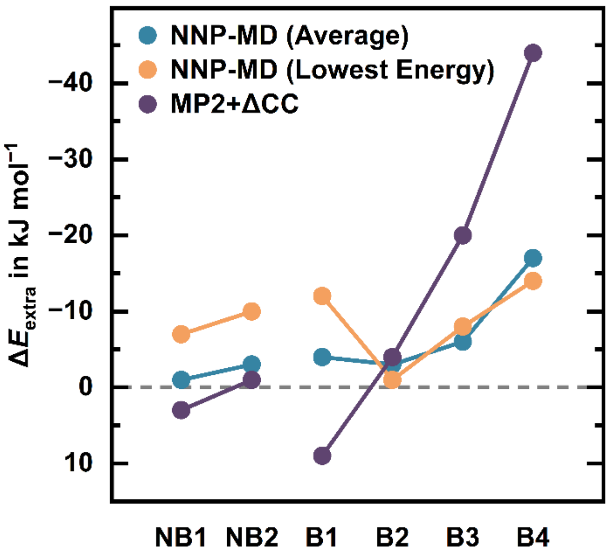

Figure S6: Extra-stabilization energies in kJ mol^{-1} for BAS pairs in H-MFI as obtained from average MLIP-MD adsorption energies, lowest MLIP-MD adsorption energies, and MP2:(PBE+D2)+ΔCC adsorption energies. Note that the average of some points is lower than the lowest energy value. The values shown are extra-stabilization energies and therefore do not represent absolute energies. Extra-stabilization energies effectively quantify the energy difference between structures containing two water molecules and the corresponding reference structures with one and zero water molecules. Consequently, these values can appear lower than the difference between the average MD energy and the lowest energy structure. Absolute energies are minimized for the lowest-energy configurations and are typically lower by 10-15 eV in MFI.

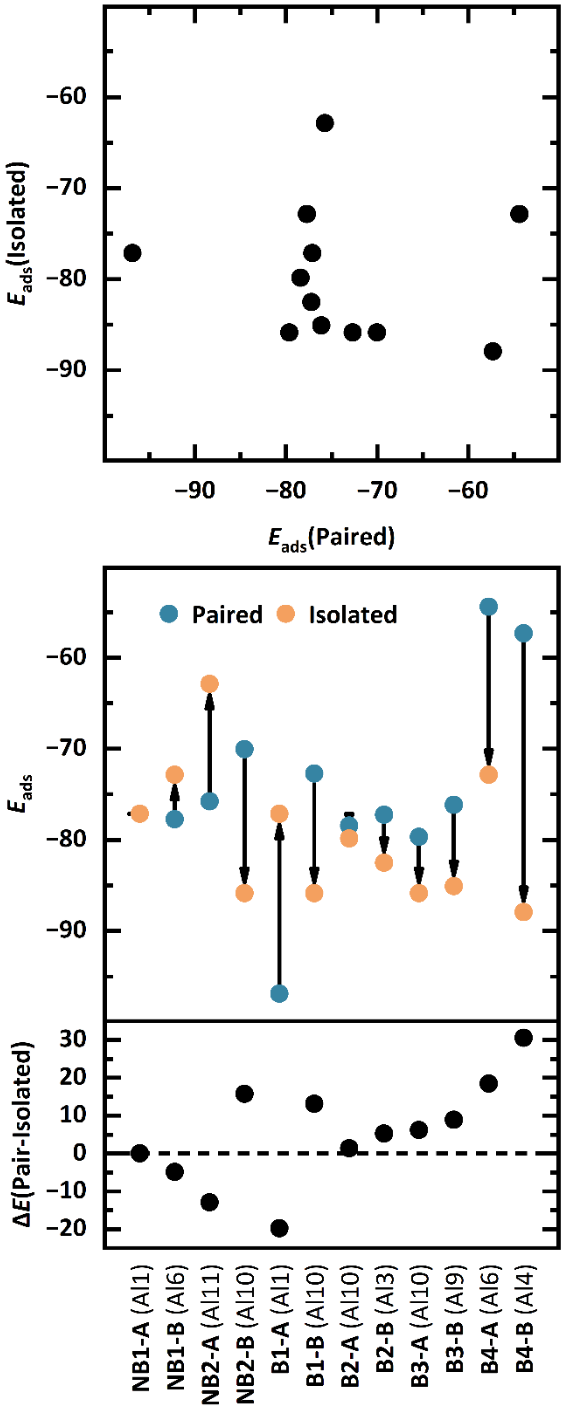

Figure S7: MP2:(PBE+D2)+ΔCC adsorption energies (E_{ads}) in kJ mol^{-1} for the adsorption of one water molecule at each site in a BAS pair (1 H_2O-A and 1 H_2O-B) as well as at the corresponding isolated BAS at the same framework position, see Tables S1, S8, and S10.

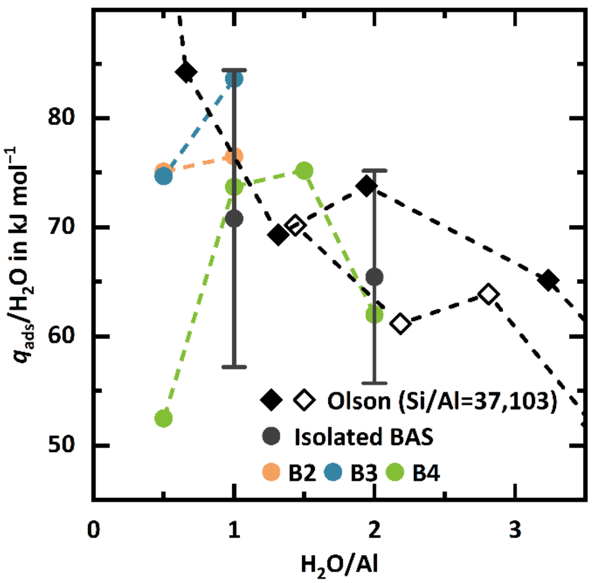

Figure S8: Experimental adsorption heats (q_{ads}) in kJ mol^{-1} per H_2O and per Al measured by Olson and co-workers.[47] MP2+ΔCC adsorption enthalpies at 298 K for isolated BAS (black bars), as well as for BAS pairs B2, B3, and B4 which show the negative $\Delta E_{\mathrm{extra}}$ values, see Table S1, S8, and S10.

Figures S9-S14 present cluster models used with MP2 and CCSD(T) in MP2:PBE+D2 structure optimizations and MP2:(PBE+D2)+ΔCC single-point energy calculations. Figures S15-S20 show the water adsorption structures optimized with MP2:PBE+D2.

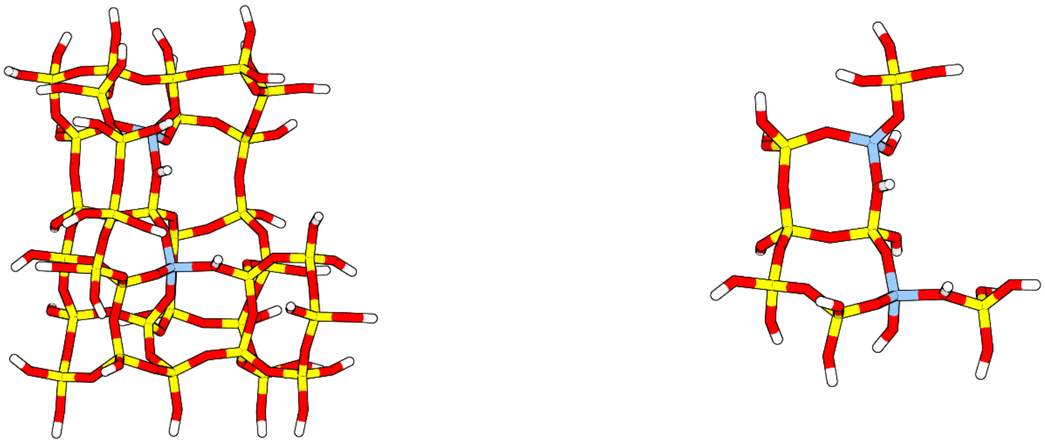

Figure S9: Clusters used for BAS pair 3 (B1). Left: Cluster used with MP2 in MP2:(PBE+D2)+ΔCC single-point energy calculations. Right: Cluster used with MP2 in MP2:PBE+D2 structure optimizations and with CCSD(T) in MP2:(PBE+D2)+ΔCC single-point energy calculations.

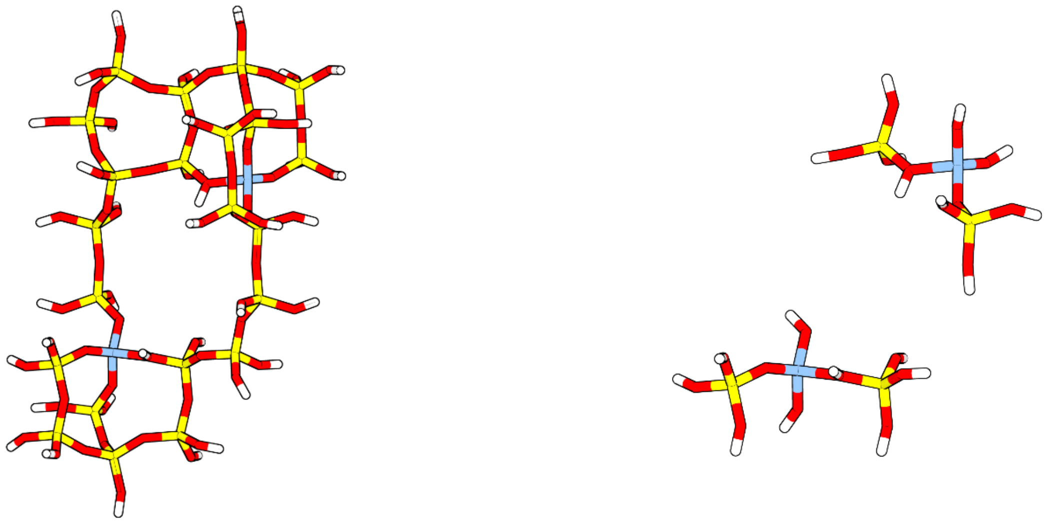

Figure S10: Clusters used for BAS pair 4 (NB1). Left: Cluster used with MP2 in MP2:(PBE+D2)+ΔCC single-point energy calculations. Right: Cluster used with MP2 in MP2:PBE+D2 structure optimizations and with CCSD(T) in MP2:(PBE+D2)+ΔCC single-point energy calculations.

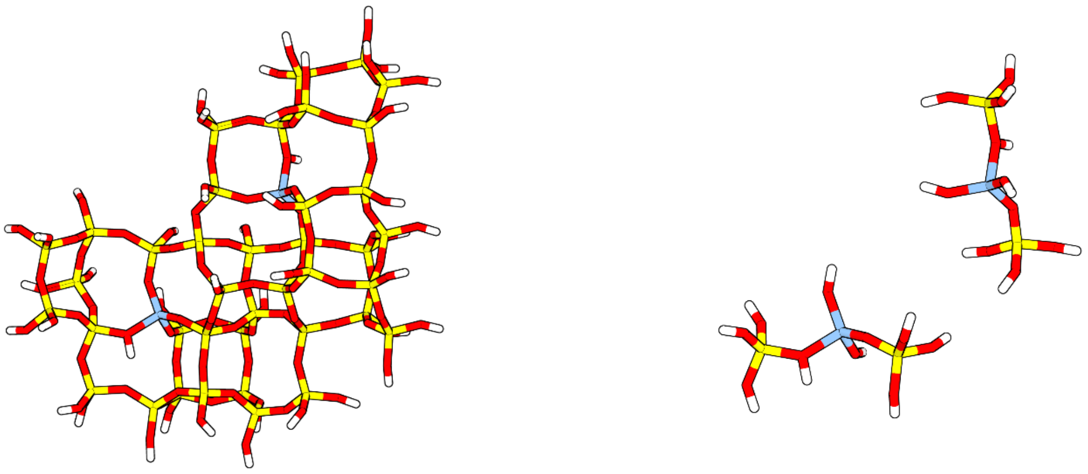

Figure S11: Clusters used for BAS pair 5 (NB2). Left: Cluster used with MP2 in MP2:(PBE+D2)+ΔCC single-point energy calculations. Right: Cluster used with MP2 in MP2:PBE+D2 structure optimizations and with CCSD(T) in MP2:(PBE+D2)+ΔCC single-point energy calculations.

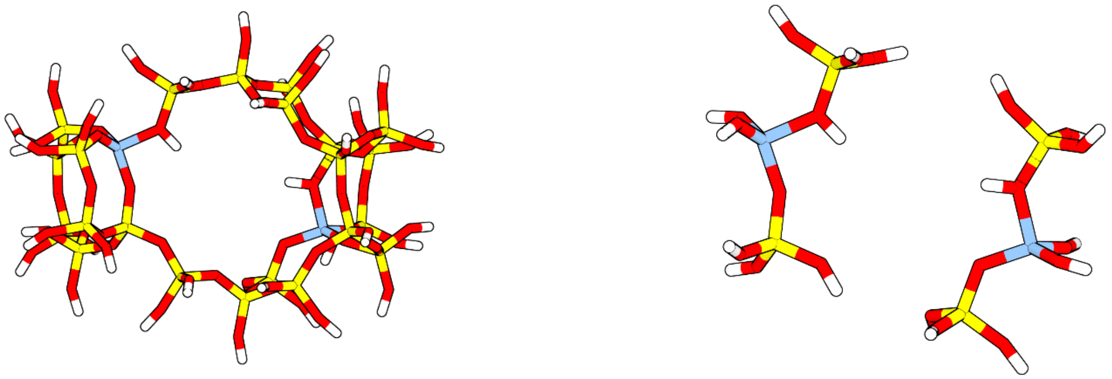

Figure S12: Clusters used for BAS pair 13 (B3). Left: Cluster used with MP2 in MP2:(PBE+D2)+ΔCC single-point energy calculations. Right: Cluster used with MP2 in MP2:PBE+D2 structure optimizations and with CCSD(T) in MP2:(PBE+D2)+ΔCC single-point energy calculations.

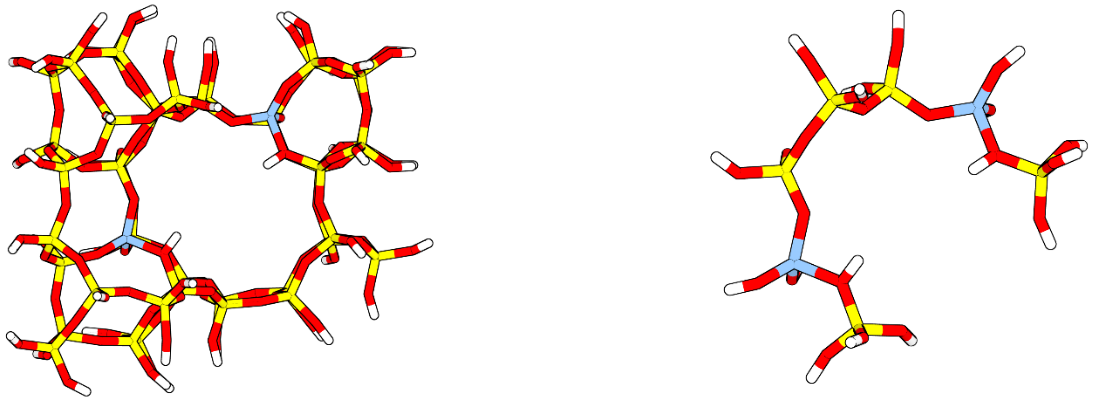

15-mp2

15-cc

Figure S13: Clusters used for BAS pair 15 (B2). Left: Cluster used with MP2 in MP2:(PBE+D2)+ΔCC single-point energy calculations. Right: Cluster used with MP2 in MP2:PBE+D2 structure optimizations and with CCSD(T) in MP2:(PBE+D2)+ΔCC single-point energy calculations.

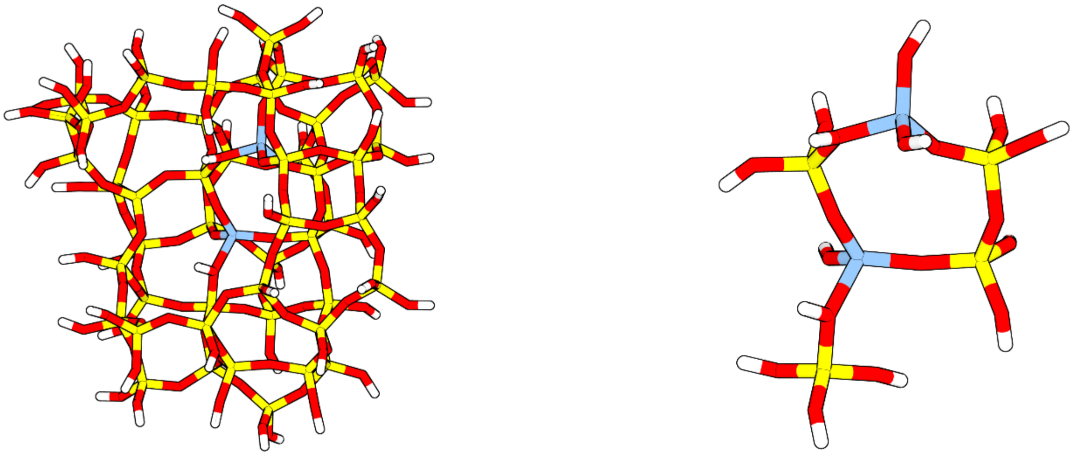

Figure S14: Clusters used for BAS pair Sci (B4). Left: Cluster used with MP2 in MP2:(PBE+D2)+ΔCC single-point energy calculations. Right: Cluster used with MP2 in MP2:PBE+D2 structure optimizations and with CCSD(T) in MP2:(PBE+D2)+ΔCC single-point energy calculations.

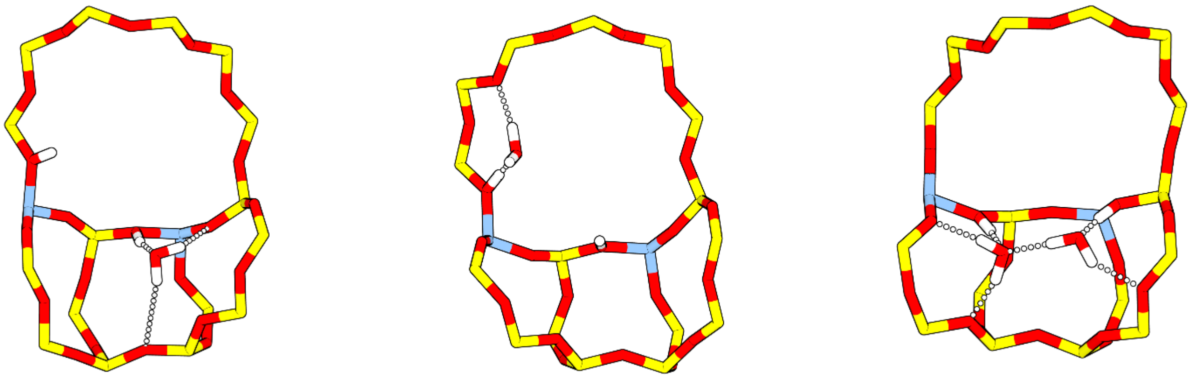

Figure S15: Water adsorption structures for BAS pair 3 (B1) optimized with MP2:PBE+D2: 1 H_2O-A (left), 1 H_2O-B (middle), and 2 H_2O (right).

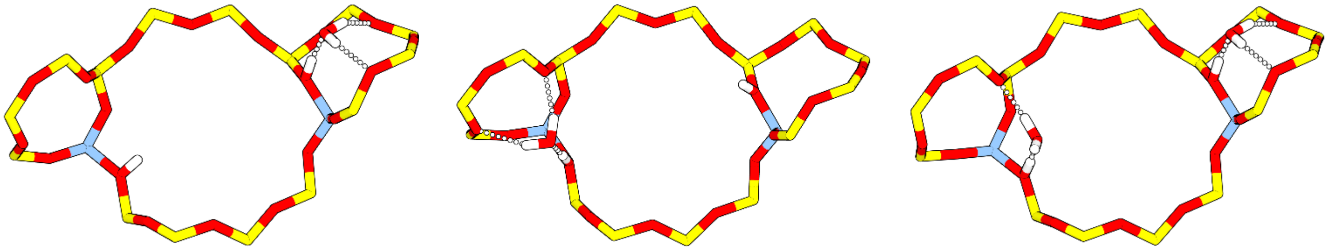

Figure S16: Water adsorption structures for BAS pair 4 (NB1) optimized with MP2:PBE+D2: 1 H_2O-A (left), 1 H_2O-B (middle), and 2 H_2O (right).

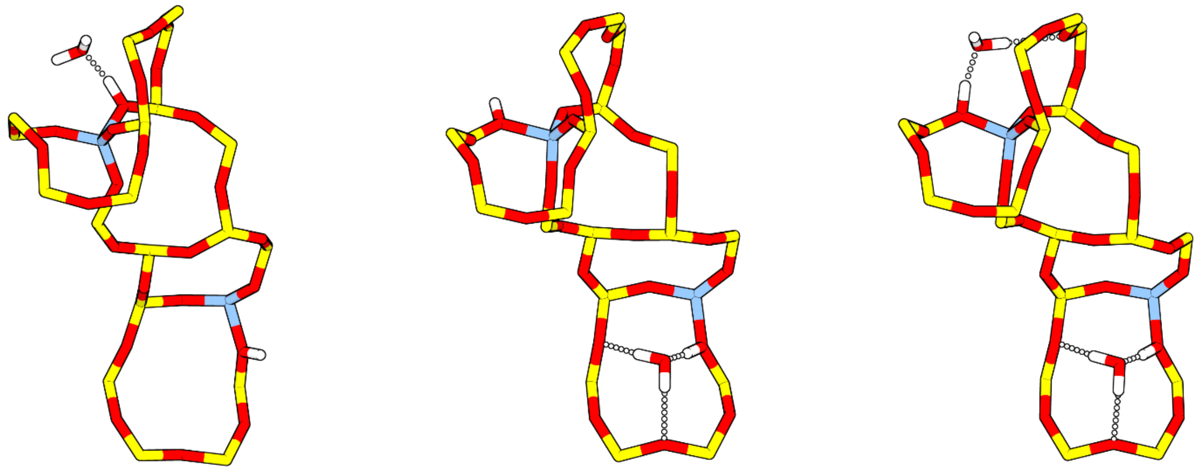

Figure S17: Water adsorption structures for BAS pair 5 (NB2) optimized with MP2:PBE+D2: 1 H_2O-A (left), 1 H_2O-B (middle), and 2 H_2O (right).

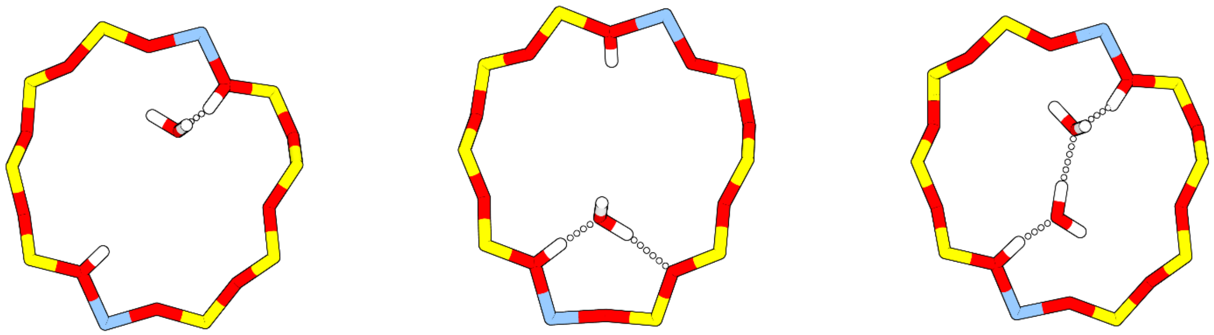

Figure S18: Water adsorption structures for BAS pair 13 (B3) optimized with MP2:PBE+D2: 1 H_2O-A (left), 1 H_2O-B (middle), and 2 H_2O (right).

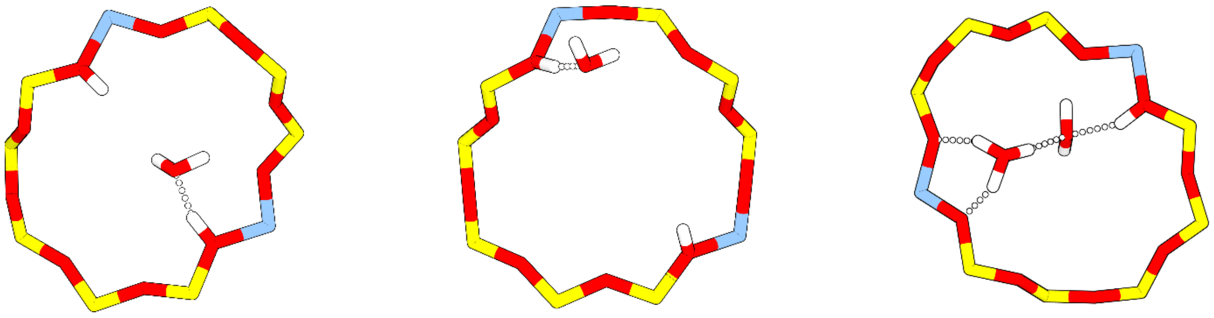

Figure S19: Water adsorption structures for BAS pair 15 (B2) optimized with MP2:PBE+D2: 1 H_2O-A (left), 1 H_2O-B (middle), and 2 H_2O (right).

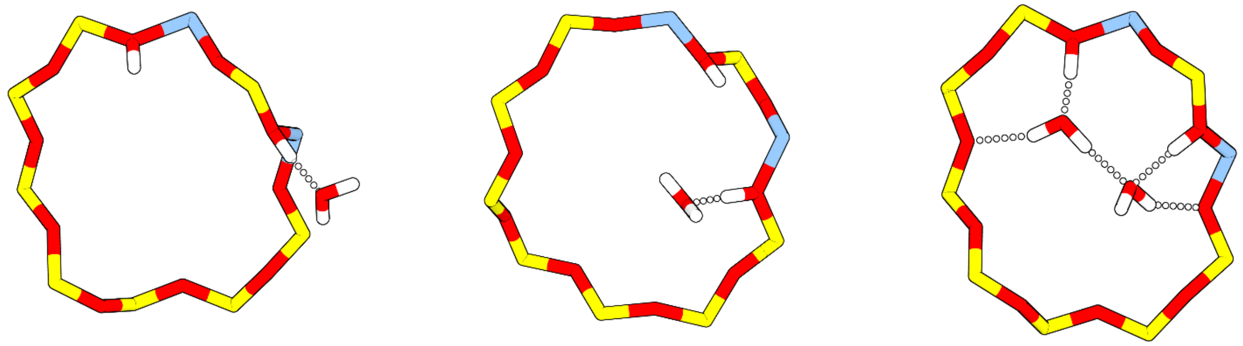

Figure S20: Water adsorption structures for BAS pair Sci (B4) optimized with MP2:PBE+D2: 1 H_2O-A (left), 1 H_2O-B (middle), and 2 H_2O (right).

S3.3 Water Adsorption at BAS Pairs in H-FAU

Tables S13 and S14 show the PBE+D2 and MP2:(PBE+D2)+ΔCC results for H-FAU, respectively. Figures S21-S22 show the employed cluster models and Figures S23-S24 show the water adsorption structures optimized with MP2:PBE+D2.

Table S13: For H-FAU, PBE+D2 adsorption energies (E_{ads}) and enthalpies at 298 K (H_{ads}) with zero-point vibrational energies (ΔZPE) and thermal contributions ($\Delta H_{0K\rightarrow 298K}$) as well as extra-stabilization energies (ΔE_{extra}) and enthalpies (ΔH_{extra}) in kJ mol^{-1}, see Eq. 1. Structures optimized with PBE+D2.

Pair ID	Loading	ΔZPE	$\Delta H_{0K\rightarrow 298K}$	E_{ads}	E_{ads}/H_2O	H_{ads}	H_{ads}/H_2O	ΔE_{extra}	ΔH_{extra}
FAU-1	1 H_2O-A	7.7	–3.6	–83.0	–83.0	–78.9	–78.9	–	–
(B-FAU)	1 H_2O-B	8.5	–4.6	–87.5	–87.5	–83.6	–83.6	–	–
	2 H_2O	19.0	–9.1	–181.8	–90.9	–172.0	–86.0	–11.3	–9.4
FAU-2	1 H_2O-A	7.4	–6.4	–103.1	–103.1	–102.0	–102.0	–	–
	1 H_2O-B	6.0	–5.8	–98.3	–98.3	–98.2	–98.2	–	–
	2 H_2O	15.5	–11.9	–196.8	–98.4	–193.2	–96.6	4.6	7.1

Table S14: For H-FAU, MP2:(PBE+D2)+ΔCC adsorption energies (E_{ads}) and enthalpies at 298 K (H_{ads}) with corrections from MP2 (ΔMP2) and CCSD(T) (ΔCC) as well as extra-stabilization energies (ΔE_{extra}) and enthalpies (ΔH_{extra}) in kJ mol^{-1}, see Eq. 1. Structures optimized with MP2:PBE+D2. Enthalpies calculated with zero-point vibrational energies (ΔZPE) and thermal contributions ($\Delta H_{0K\rightarrow 298K}$) from PBE+D2, see Table S13.

Pair ID	Loading	ΔMP2	ΔMP2 / H_2O	ΔCC	ΔCC / H_2O	E_{ads}	E_{ads}/ H_2O	H_{ads}	H_{ads}/ H_2O	ΔE_{extra}	ΔH_{extra}
FAU-1 (B-FAU)	1 H_2O-A	10.0	10.0	0.8	0.8	−70.5	−70.5	−66.5	−66.5		
	1 H_2O-B	10.6	10.6	0.9	0.9	−74.3	−74.3	−70.4	−70.4		
	2 H_2O	21.4	10.7	1.9	0.9	−155.8	−77.9	−146.0	−73.0	−11.1	−9.2
FAU-2	1 H_2O-A	19.5	19.5	2.1	2.1	−79.0	−79.0	−78.0	−78.0		
	1 H_2O-B	19.7	19.7	2.3	2.3	−78.8	−78.8	−78.7	−78.7		
	2 H_2O	30.1	15.0	3.6	1.8	−157.8	−78.9	−154.2	−77.1	0.0	2.5

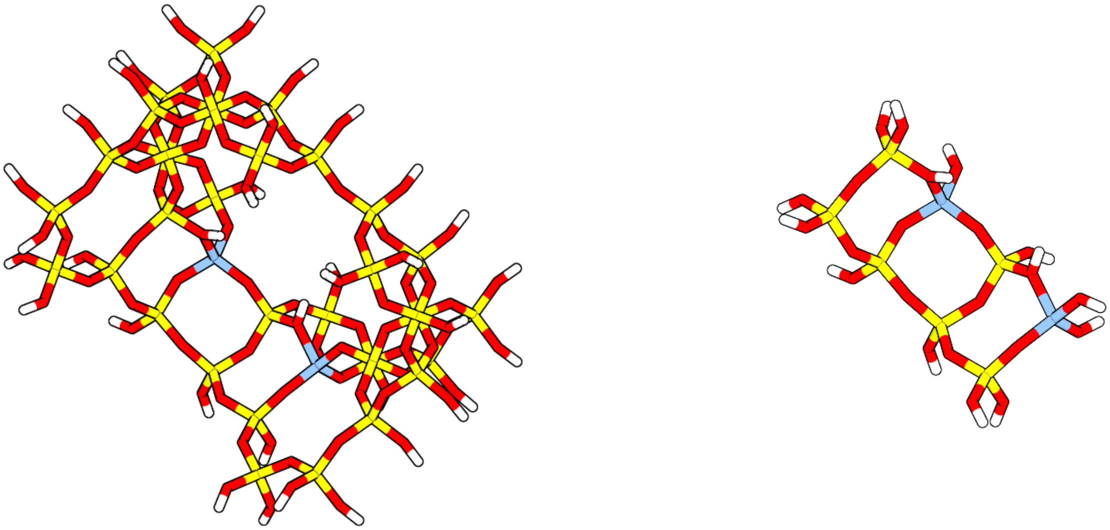

Figure S21: Clusters used for BAS pair FAU-1 (B-FAU). Left: Cluster used with MP2 in MP2:(PBE+D2)+ΔCC single-point energy calculations. Right: Cluster used with MP2 in MP2:PBE+D2 structure optimizations and with CCSD(T) in MP2:(PBE+D2)+ΔCC single-point energy calculations.

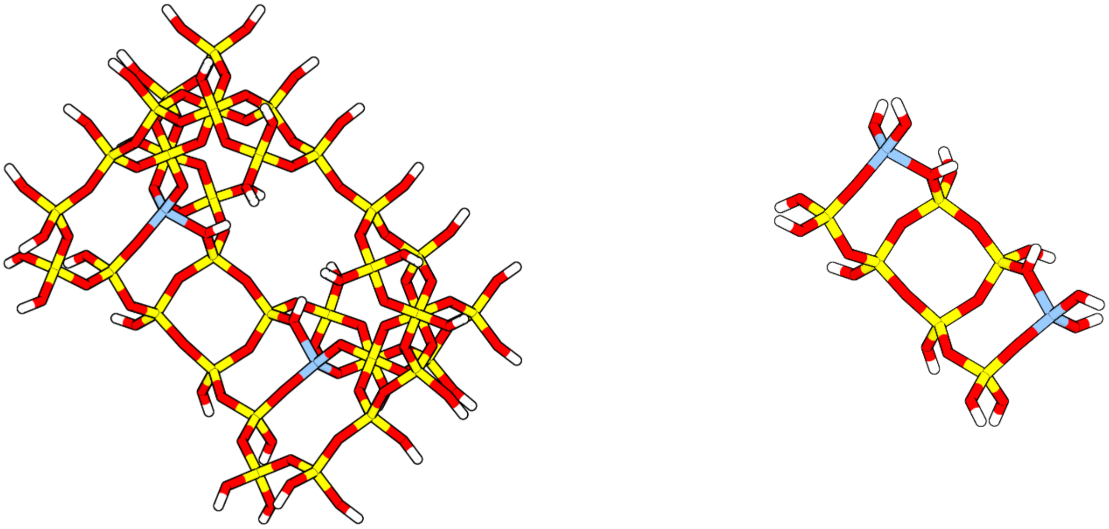

Figure S22: Clusters used for BAS pair FAU-2. Left: Cluster used with MP2 in MP2:(PBE+D2)+ΔCC single-point energy calculations. Right: Cluster used with MP2 in MP2:PBE+D2 structure optimizations and with CCSD(T) in MP2:(PBE+D2)+ΔCC single-point energy calculations.

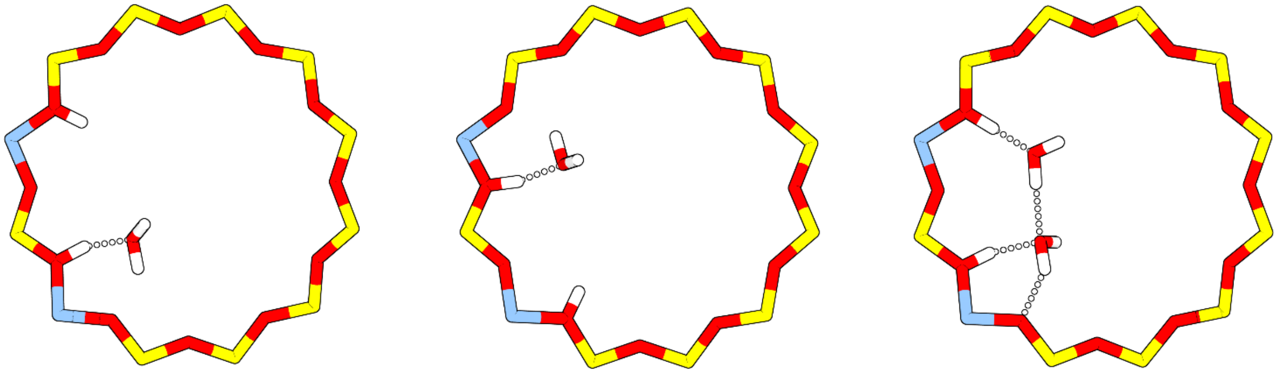

Figure S23: Water adsorption structures for BAS pair FAU-1 (B-FAU) optimized with MP2:PBE+D2: 1 H_2O-A (left), 1 H_2O-B (middle), and 2 H_2O (right).

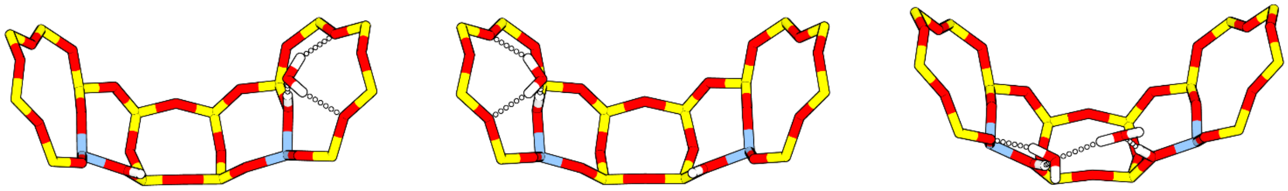

Figure S24: Water adsorption structures for BAS pair FAU-2 optimized with MP2:PBE+D2: 1 H_2O-A (left), 1 H_2O-B (middle), and 2 H_2O (right).

S3.4 Water Adsorption at BAS Pairs in H-CHA

Tables S15 and S16 show the PBE+D2 and MP2:(PBE+D2)+ΔCC results for H-CHA, respectively. Figures S25-S26 show the employed cluster models and Figures S27-S28 show the water adsorption structures optimized with MP2:PBE+D2.

Table S15: For H-CHA, PBE+D2 adsorption energies (E_{ads}) and enthalpies at 298 K (H_{ads}) with zero-point vibrational energies (ΔZPE) and thermal contributions ($\Delta H_{0K\rightarrow 298K}$) as well as extra-stabilization energies (ΔE_{extra}) and enthalpies (ΔH_{extra}) in kJ mol^{-1}, see Eq. 1. Structures optimized with PBE+D2.

Pair ID	Loading	ΔZPE	$\Delta H_{0K\rightarrow 298K}$	E_{ads}	E_{ads}/H_2O	H_{ads}	H_{ads}/H_2O	ΔE_{extra}	ΔH_{extra}
CHA-1	1 H_2O-A	7.5	–6.2	–102.5	–102.5	–101.2	–101.2	–	–
	1 H_2O-B	7.0	–6.5	–110.9	–110.9	–110.4	–110.4	–	–
	2 H_2O	17.1	–9.2	–187.7	–93.8	–179.7	–89.9	25.7	31.9
CHA-2	1 H_2O-A	7.7	–4.1	–109.0	–109.0	–105.4	–105.4	–	–
	1 H_2O-B	7.9	–4.8	–104.0	–104.0	–100.9	–100.9	–	–
	2 H_2O	15.2	–10.8	–209.6	–104.8	–205.2	–102.6	3.5	1.2

Table S16: For H-CHA, MP2:(PBE+D2)+ΔCC adsorption energies (E_{ads}) and enthalpies at 298 K (H_{ads}) with corrections from MP2 (ΔMP2) and CCSD(T) (ΔCC) as well as extra-stabilization energies (ΔE_{extra}) and enthalpies (ΔH_{extra}) in kJ mol^{-1}, see Eq. 1. Structures optimized with MP2:PBE+D2. Enthalpies calculated with zero-point vibrational energies (ΔZPE) and thermal contributions ($\Delta H_{0K\rightarrow 298K}$) from PBE+D2, see Table S15.

Pair ID	Loading	ΔMP2	ΔMP2/ H_2O	ΔCC	ΔCC/ H_2O	E_{ads}	E_{ads}/ H_2O	H_{ads}	H_{ads}/ H_2O	ΔE_{extra}	ΔH_{extra}
CHA-1	1 H_2O-A	12.4	12.4	1.8	1.8	–86.2	–86.2	–84.9	–84.9		
	1 H_2O-B	20.1	20.1	1.6	1.6	–87.2	–87.2	–86.7	–86.7		
	2 H_2O	30.0	15.0	1.7	0.8	–153.8	–76.9	–145.9	–72.9	19.6	25.8
CHA-2	1 H_2O-A	8.1	8.1	0.4	0.4	–99.3	–99.3	–95.7	–95.7		
	1 H_2O-B	16.2	16.2	1.1	1.1	–85.4	–85.4	–82.3	–82.3		
	2 H_2O	28.5	14.2	2.3	1.2	–175.6	–87.8	–171.2	–85.6	9.1	6.8

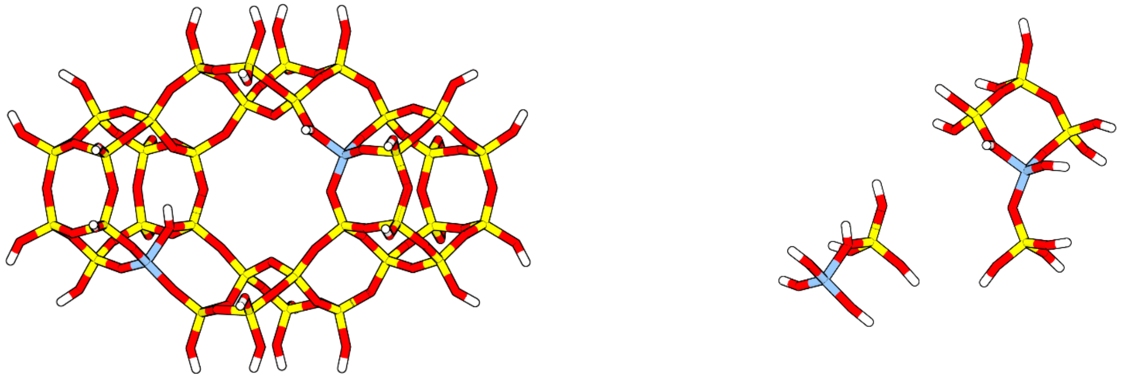

Figure S25: Clusters used for BAS pair CHA-1. Left: Cluster used with MP2 in MP2:(PBE+D2)+ΔCC single-point energy calculations. Right: Cluster used with MP2 in MP2:PBE+D2 structure optimizations and with CCSD(T) in MP2:(PBE+D2)+ΔCC single-point energy calculations.

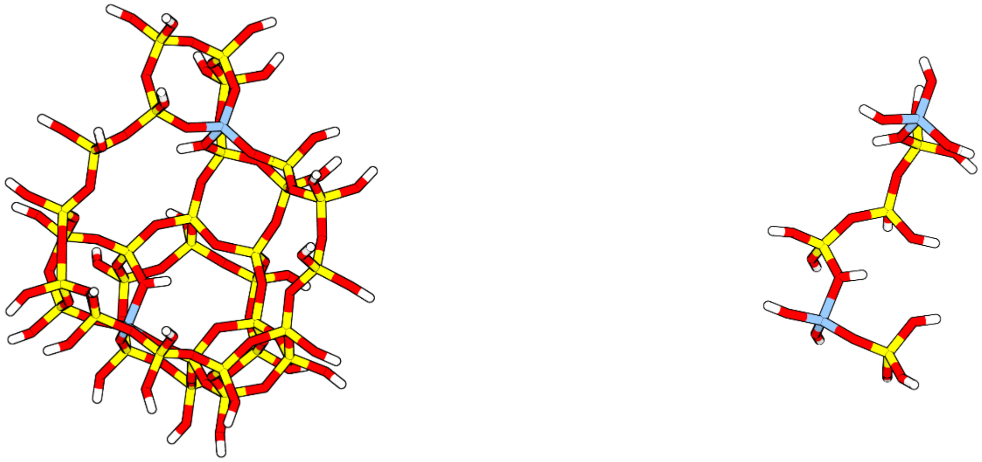

Figure S26: Clusters used for BAS pair CHA-2. Left: Cluster used with MP2 in MP2:(PBE+D2)+ΔCC single-point energy calculations. Right: Cluster used with MP2 in MP2:PBE+D2 structure optimizations and with CCSD(T) in MP2:(PBE+D2)+ΔCC single-point energy calculations.

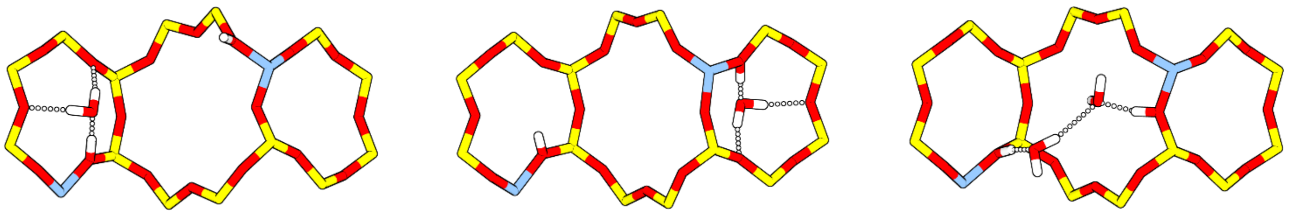

Figure S27: Water adsorption structures for BAS pair CHA-1 optimized with MP2:PBE+D2: 1 H_2O-A (left), 1 H_2O-B (middle), and 2 H_2O (right).

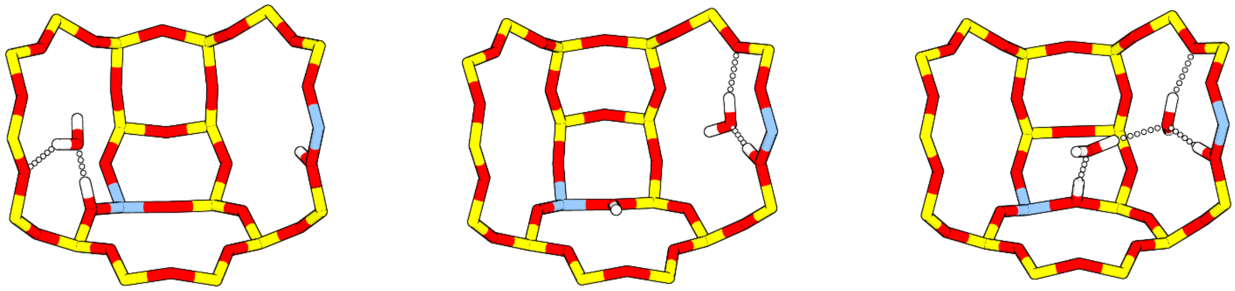

Figure S28: Water adsorption structures for BAS pair CHA-2 optimized with MP2:PBE+D2: 1 H_2O-A (left), 1 H_2O-B (middle), and 2 H_2O (right).

References:

1. Baerlocher, C.; McCusker, L. B. Database of Zeolite Structures. http://www.iza-structure.org/databases/ (accessed 05.06.2020).
2. Erlebach, A.; Šípka, M.; Saha, I.; Nachtigall, P.; Heard, C. J.; Grajciar, L., A reactive neural network framework for water-loaded acidic zeolites. *Nat. Commun.* **2024,** *15*, 4215, https://doi.org/10.1038/s41467-024-48609-2.
3. Sun, J.; Remsing, R. C.; Zhang, Y.; Sun, Z.; Ruzsinszky, A.; Peng, H.; Yang, Z.; Paul, A.; Waghmare, U.; Wu, X., SCAN: An efficient density functional yielding accurate structures and energies of diversely-bonded materials. *arXiv* **2015**, https://doi.org/10.48550/arXiv.1511.01089.
4. Grimme, S.; Hansen, A.; Brandenburg, J. G.; Bannwarth, C., Dispersion-Corrected Mean-Field Electronic Structure Methods. *Chem. Rev.* **2016,** *116*, 5105-5154, https://doi.org/10.1021/acs.chemrev.5b00533.
5. Willimetz, D.; Erlebach, A.; Heard, C. J.; Grajciar, L., 27Al NMR chemical shifts in zeolite MFI via machine learning acceleration of structure sampling and shift prediction. *Digit. Discov.* **2025,** *4*, 275-288, https://doi.org/10.1039/D4DD00306C.
6. Windeck, H.; Berger, F.; Sauer, J., Chemically accurate predictions for water adsorption on Brønsted sites of zeolite H-MFI. *Phys. Chem. Chem. Phys.* **2024,** *26*, 23588-23599, https://doi.org/10.1039/D4CP02851A.
7. Willimetz, D.; Grajciar, L., A Simple and Scalable Kernel Density Approach for Reliable Uncertainty Quantification in Atomistic Machine Learning. *J. Phys. Chem. Lett.* **2025,** *16*, 11081-11086, https://doi.org/10.1021/acs.jpclett.5c02595.
8. Hjorth Larsen, A.; Jørgen Mortensen, J.; Blomqvist, J.; Castelli, I. E.; Christensen, R.; Dułak, M.; Friis, J.; Groves, M. N.; Hammer, B.; Hargus, C.; Hermes, E. D.; Jennings, P. C.; Bjerre Jensen, P.; Kermode, J.; Kitchin, J. R.; Leonhard Kolsbjerg, E.; Kubal, J.; Kaasbjerg, K.; Lysgaard, S.; Bergmann Maronsson, J.; Maxson, T.; Olsen, T.; Pastewka, L.; Peterson, A.; Rostgaard, C.; Schiøtz, J.; Schütt, O.; Strange, M.; Thygesen, K. S.; Vegge, T.; Vilhelmsen, L.; Walter, M.; Zeng, Z.; Jacobsen, K. W., The atomic simulation environment—a Python library for working with atoms. *J. Phys.: Condens. Matter* **2017,** *29*, 273002, https://doi.org/10.1088/1361-648X/aa680e.
9. Saha, I.; Grajciar, L.; Willimetz, D., CRISP: Enhancing ASE Workflows with Advanced Molecular Simulation Post-Processing. *ChemRxiv* **2025**, https://doi.org/10.26434/chemrxiv-2025-dht8g.
10. Chib, S.; Greenberg, E., Understanding the Metropolis-Hastings Algorithm. *The American Statistician* **1995,** *49*, 327-335, https://doi.org/10.1080/00031305.1995.10476177.
11. Eckstein, S.; Hintermeier, P. H.; Zhao, R.; Baráth, E.; Shi, H.; Liu, Y.; Lercher, J. A., Influence of Hydronium Ions in Zeolites on Sorption. *Angew. Chem. Int. Ed.* **2019,** *58*, 3450-3455, https://doi.org/10.1002/anie.201812184.
12. Grabowski, S. J., Hydrogen Bond and Other Lewis Acid–Lewis Base Interactions as Preliminary Stages of Chemical Reactions. *Molecules* **2020,** *25*, 4668, https://doi.org/10.3390/molecules25204668.
13. Arunan, E.; Desiraju, G. R.; Klein, R. A.; Sadlej, J.; Scheiner, S.; Alkorta, I.; Clary, D. C.; Crabtree, R. H.; Dannenberg, J. J.; Hobza, P.; Kjaergaard, H. G.; Legon, A. C.; Mennucci, B.; Nesbitt, D. J., Definition of the hydrogen bond (IUPAC Recommendations 2011). *Pure Appl. Chem.* **2011,** *83*, 1637-1641, https://doi.org/doi:10.1351/PAC-REC-10-01-02.
14. Sokal, A., Monte Carlo Methods in Statistical Mechanics: Foundations and New Algorithms. In *Functional Integration: Basics and Applications*, DeWitt-Morette, C.; Cartier, P.; Folacci, A., Eds. Springer US: Boston, MA, 1997; pp 131-192.
15. Upton, G. J. G., Fisher's Exact Test. *Journal of the Royal Statistical Society: Series A (Statistics in Society)* **1992,** *155*, 395-402, https://doi.org/10.2307/2982890.
16. Perdew, J. P.; Burke, K.; Ernzerhof, M., Generalized Gradient Approximation Made Simple. *Phys. Rev. Lett.* **1996,** *77*, 3865-3868, https://doi.org/10.1103/PhysRevLett.77.3865.
17. Grimme, S., Semiempirical GGA-type density functional constructed with a long-range dispersion correction. *J. Comput. Chem.* **2006,** *27*, 1787-1799, https://doi.org/10.1002/jcc.20495.

18. Kerber, T.; Sierka, M.; Sauer, J., Application of semiempirical long-range dispersion corrections to periodic systems in density functional theory. *J. Comput. Chem.* **2008,** *29*, 2088-2097, https://doi.org/10.1002/jcc.21069.
19. Kresse, G.; Furthmüller, J., Efficiency of ab-initio total energy calculations for metals and semiconductors using a plane-wave basis set. *Comput. Mater. Sci.* **1996,** *6*, 15-50, https://doi.org/10.1016/0927-0256(96)00008-0.
20. Kresse, G.; Furthmüller, J., Efficient iterative schemes for ab initio total-energy calculations using a plane-wave basis set. *Phys. Rev. B* **1996,** *54*, 11169-11186, https://doi.org/10.1103/PhysRevB.54.11169.
21. Kresse, G.; Hafner, J., Ab initio molecular dynamics for liquid metals. *Phys. Rev. B* **1993,** *47*, 558-561, https://doi.org/10.1103/PhysRevB.47.558.
22. Kresse, G.; Joubert, D., From ultrasoft pseudopotentials to the projector augmented-wave method. *Phys. Rev. B* **1999,** *59*, 1758-1775, https://doi.org/10.1103/PhysRevB.59.1758.
23. Ren, Q.; Rybicki, M.; Sauer, J., Interaction of C3–C5 Alkenes with Zeolitic Brønsted Sites: π-Complexes, Alkoxides, and Carbenium Ions in H-FER. *J. Phys. Chem. C* **2020,** *124*, 10067-10078, https://doi.org/10.1021/acs.jpcc.0c03061.
24. Rybicki, M.; Sauer, J., Ab Initio Prediction of Proton Exchange Barriers for Alkanes at Brønsted Sites of Zeolite H-MFI. *J. Am. Chem. Soc.* **2018,** *140*, 18151-18161, https://doi.org/10.1021/jacs.8b11228.
25. Berger, F.; Rybicki, M.; Sauer, J., Molecular Dynamics with Chemical Accuracy—Alkane Adsorption in Acidic Zeolites. *ACS Catal.* **2023,** *13*, 2011-2024, https://doi.org/10.1021/acscatal.2c05493.
26. Berger, F.; Rybicki, M.; Sauer, J., Adsorption and cracking of propane by zeolites of different pore size. *J. Catal.* **2021,** *395*, 117-128, https://doi.org/10.1016/j.jcat.2020.12.008.
27. Rehak, F. R.; Piccini, G.; Alessio, M.; Sauer, J., Including dispersion in density functional theory for adsorption on flat oxide surfaces, in metal–organic frameworks and in acidic zeolites. *Phys. Chem. Chem. Phys.* **2020,** *22*, 7577-7585, https://doi.org/10.1039/D0CP00394H.
28. Sauer, J., Ab Initio Calculations for Molecule–Surface Interactions with Chemical Accuracy. *Acc. Chem. Res.* **2019,** *52*, 3502-3510, https://doi.org/10.1021/acs.accounts.9b00506.
29. Sierka, M.; Sauer, J., Finding transition structures in extended systems: A strategy based on a combined quantum mechanics–empirical valence bond approach. *J. Chem. Phys.* **2000,** *112*, 6983-6996, https://doi.org/10.1063/1.481296.
30. Eichler, U.; Kölmel, C. M.; Sauer, J., Combining ab initio techniques with analytical potential functions for structure predictions of large systems: Method and application to crystalline silica polymorphs. *J. Comput. Chem.* **1997,** *18*, 463-477, https://doi.org/10.1002/(SICI)1096-987X(199703)18:4<463::AID-JCC2>3.0.CO;2-R.
31. Tuma, C.; Sauer, J., A hybrid MP2/planewave-DFT scheme for large chemical systems: proton jumps in zeolites. *Chem. Phys. Lett.* **2004,** *387*, 388-394, https://doi.org/10.1016/j.cplett.2004.02.056.
32. Alessio, M.; Bischoff, F. A.; Sauer, J., Chemically accurate adsorption energies for methane and ethane monolayers on the MgO(001) surface. *Phys. Chem. Chem. Phys.* **2018,** *20*, 9760-9769, https://doi.org/10.1039/C7CP08083B.
33. Møller, C.; Plesset, M. S., Note on an Approximation Treatment for Many-Electron Systems. *Phys. Rev.* **1934,** *46*, 618-622, https://doi.org/10.1103/PhysRev.46.618.
34. Pople, J. A.; Binkley, J. S.; Seeger, R., Theoretical models incorporating electron correlation. *Int. J. Quantum Chem.* **1976,** *10*, 1-19, https://doi.org/10.1002/qua.560100802.
35. Raghavachari, K.; Trucks, G. W.; Pople, J. A.; Head-Gordon, M., A fifth-order perturbation comparison of electron correlation theories. *Chem. Phys. Lett.* **1989,** *157*, 479-483, https://doi.org/10.1016/S0009-2614(89)87395-6.
36. Urban, M.; Noga, J.; Cole, S. J.; Bartlett, R. J., Towards a full CCSDT model for electron correlation. *J. Chem. Phys.* **1985,** *83*, 4041-4046, https://doi.org/10.1063/1.449067.
37. Neese, F.; Wennmohs, F.; Becker, U.; Riplinger, C., The ORCA quantum chemistry program package. *J. Chem. Phys.* **2020,** *152*, https://doi.org/10.1063/5.0004608.

38. Weigend, F.; Ahlrichs, R., Balanced basis sets of split valence, triple zeta valence and quadruple zeta valence quality for H to Rn: Design and assessment of accuracy. *Phys. Chem. Chem. Phys.* **2005,** *7*, 3297-3305, https://doi.org/10.1039/B508541A.
39. Feyereisen, M.; Fitzgerald, G.; Komornicki, A., Use of approximate integrals in ab initio theory. An application in MP2 energy calculations. *Chem. Phys. Lett.* **1993,** *208*, 359-363, https://doi.org/10.1016/0009-2614(93)87156-W.
40. Pavošević, F.; Pinski, P.; Riplinger, C.; Neese, F.; Valeev, E. F., SparseMaps—A systematic infrastructure for reduced-scaling electronic structure methods. IV. Linear-scaling second-order explicitly correlated energy with pair natural orbitals. *J. Chem. Phys.* **2016,** *144*, https://doi.org/10.1063/1.4945444.
41. Pinski, P.; Riplinger, C.; Valeev, E. F.; Neese, F., Sparse maps—A systematic infrastructure for reduced-scaling electronic structure methods. I. An efficient and simple linear scaling local MP2 method that uses an intermediate basis of pair natural orbitals. *J. Chem. Phys.* **2015,** *143*, https://doi.org/10.1063/1.4926879.
42. Dunning, T. H., Jr., Gaussian basis sets for use in correlated molecular calculations. I. The atoms boron through neon and hydrogen. *J. Chem. Phys.* **1989,** *90*, 1007-1023, https://doi.org/10.1063/1.456153.
43. Woon, D. E.; Dunning, T. H., Jr., Gaussian basis sets for use in correlated molecular calculations. III. The atoms aluminum through argon. *J. Chem. Phys.* **1993,** *98*, 1358-1371, https://doi.org/10.1063/1.464303.
44. Jensen, F., Estimating the Hartree—Fock limit from finite basis set calculations. *Theor. Chem. Acc.* **2005,** *113*, 267-273, https://doi.org/10.1007/s00214-005-0635-2.
45. Helgaker, T.; Klopper, W.; Koch, H.; Noga, J., Basis-set convergence of correlated calculations on water. *J. Chem. Phys.* **1997,** *106*, 9639-9646, https://doi.org/10.1063/1.473863.
46. Boys, S. F.; Bernardi, F., The calculation of small molecular interactions by the differences of separate total energies. Some procedures with reduced errors. *Mol. Phys.* **1970,** *19*, 553-566, https://doi.org/10.1080/00268977000101561.
47. Olson, D. H.; Haag, W. O.; Borghard, W. S., Use of water as a probe of zeolitic properties: interaction of water with HZSM-5. *Microporous Mesoporous Mater.* **2000,** *35-36*, 435-446, https://doi.org/10.1016/S1387-1811(99)00240-1.
